# Clinical characteristics and MRI based radiomics nomograms can predict iPFS and short-term efficacy of third-generation EGFR-TKI in EGFR-mutated lung adenocarcinoma with brain metastases

**DOI:** 10.1186/s12885-024-12121-z

**Published:** 2024-03-21

**Authors:** Haoran Qi, Yichen Hou, Zhonghang Zheng, Mei Zheng, Qiang Qiao, Zihao Wang, Xiaorong Sun, Ligang Xing

**Affiliations:** 1 Department of Radiation Oncology, Shandong Cancer Hospital and Institute, Shandong First Medical University, Shandong Academy of Medical Sciences, 440 Jiyan Road, Jinan, Shandong 250117 China; 2 Department of Nuclear Medicine, Shandong Cancer Hospital and Institute, Shandong First Medical University, Shandong Academy of Medical Science, Jinan, Shandong China

**Keywords:** MRI, Radiomics, Lung adenocarcinoma, Brain metastases, Third-generation EGFR-TKI

## Abstract

**Background:**

Predicting short-term efficacy and intracranial progression-free survival (iPFS) in epidermal growth factor receptor gene mutated (EGFR-mutated) lung adenocarcinoma patients with brain metastases who receive third-generation epidermal growth factor receptor tyrosine kinase inhibitor (EGFR-TKI) therapy was of great significance for individualized treatment. We aimed to construct and validate nomograms based on clinical characteristics and magnetic resonance imaging (MRI) radiomics for predicting short-term efficacy and intracranial progression free survival (iPFS) of third-generation EGFR-TKI in EGFR-mutated lung adenocarcinoma patients with brain metastases.

**Methods:**

One hundred ninety-four EGFR-mutated lung adenocarcinoma patients with brain metastases who received third-generation EGFR-TKI treatment were included in this study from January 1, 2017 to March 1, 2023. Patients were randomly divided into training cohort and validation cohort in a ratio of 5:3. Radiomics features extracted from brain MRI were screened by least absolute shrinkage and selection operator (LASSO) regression. Logistic regression analysis and Cox proportional hazards regression analysis were used to screen clinical risk factors. Single clinical (C), single radiomics (R), and combined (C + R) nomograms were constructed in short-term efficacy predicting model and iPFS predicting model, respectively. Prediction effectiveness of nomograms were evaluated by calibration curves, Harrell’s concordance index (C-index), receiver operating characteristic (ROC) curves and decision curve analysis (DCA). Kaplan-Meier analysis was used to compare the iPFS of high and low iPFS rad-score patients in the predictive iPFS R model and to compare the iPFS of high-risk and low-risk patients in the predictive iPFS C + R model.

**Results:**

Overall response rate (ORR) was 71.1%, disease control rate (DCR) was 91.8% and median iPFS was 12.67 months (7.88–20.26, interquartile range [IQR]). There were significant differences in iPFS between patients with high and low iPFS rad-scores, as well as between high-risk and low-risk patients. In short-term efficacy model, the C-indexes of C + R nomograms in training cohort and validation cohort were 0.867 (0.835-0.900, 95%CI) and 0.803 (0.753–0.854, 95%CI), while in iPFS model, the C-indexes were 0.901 (0.874–0.929, 95%CI) and 0.753 (0.713–0.793, 95%CI).

**Conclusions:**

The third-generation EGFR-TKI showed significant efficacy in EGFR-mutated lung adenocarcinoma patients with brain metastases, and the combined line plot of C + R can be utilized to predict short-term efficacy and iPFS.

**Supplementary Information:**

The online version contains supplementary material available at 10.1186/s12885-024-12121-z.

## Background

Brain metastasis, as one of the most prevalent sites for non-small cell lung cancer (NSCLC) metastasis, is associated with a dismal prognosis, with untreated patients having a median overall survival (OS) of less than 2 months [1]. The incidence of brain metastasis in NSCLC patients with EGFR mutation is approximately 50%, significantly higher than that in wild-type patients [2–4]. Targeted therapy with EGFR-TKI has substantially improved the prognosis of EGFR-mutated NSCLC patients with brain metastasis [5]. The third-generation EGFR-TKIs, such as osimertinib, almonertinib, and furmonertinib, exhibit superior blood-brain barrier permeability, selectivity, and safety compared to their predecessors [6]. These agents have become the standard treatment for EGFR-mutated NSCLC patients with brain metastases who have developed resistance to previous EGFR-TKIs due to T790M mutation or for untreated patients receiving first-line therapy [7]. However, not all patients achieve satisfactory outcomes following treatment with third-generation EGFR-TKIs. Approximately 20% of patients experience resistance upon initial administration. Furthermore, even if an initial response is observed when using these agents, the timing of disease progression varies [8]. Therefore, accurate prediction of therapeutic efficacy and timely adjustment of treatment plans are crucial. Previous studies have demonstrated a significant correlation between baseline clinical characteristics and the effectiveness of EGFR-TKI therapy [9]. Radiomics enables extraction of high-throughput quantitative features from medical images such as computed tomography (CT), magnetic resonance imaging (MRI), and positron emission tomography-computed tomography (PET-CT), which may be imperceptible to human observers [10]. Nomograms serve as predictive tools by integrating multiple decisive variables related to efficacy and prognosis [11]. Nomograms incorporating radiomics and baseline clinical characteristics have been extensively utilized in medical and oncology research [12–14]. This study aims to establish and validate radiomics models based on clinical features and MRI scans of EGFR-mutated lung adenocarcinoma patients with brain metastases prior to receiving third-generation EGFR-TKI treatment, in order to predict short-term efficacy and iPFS, helping clinicians to identify high-risk patients in a timely manner and adjust diagnostic and treatment strategies accordingly.

## Materials and methods

### Patients and design

**Fig. 1:**
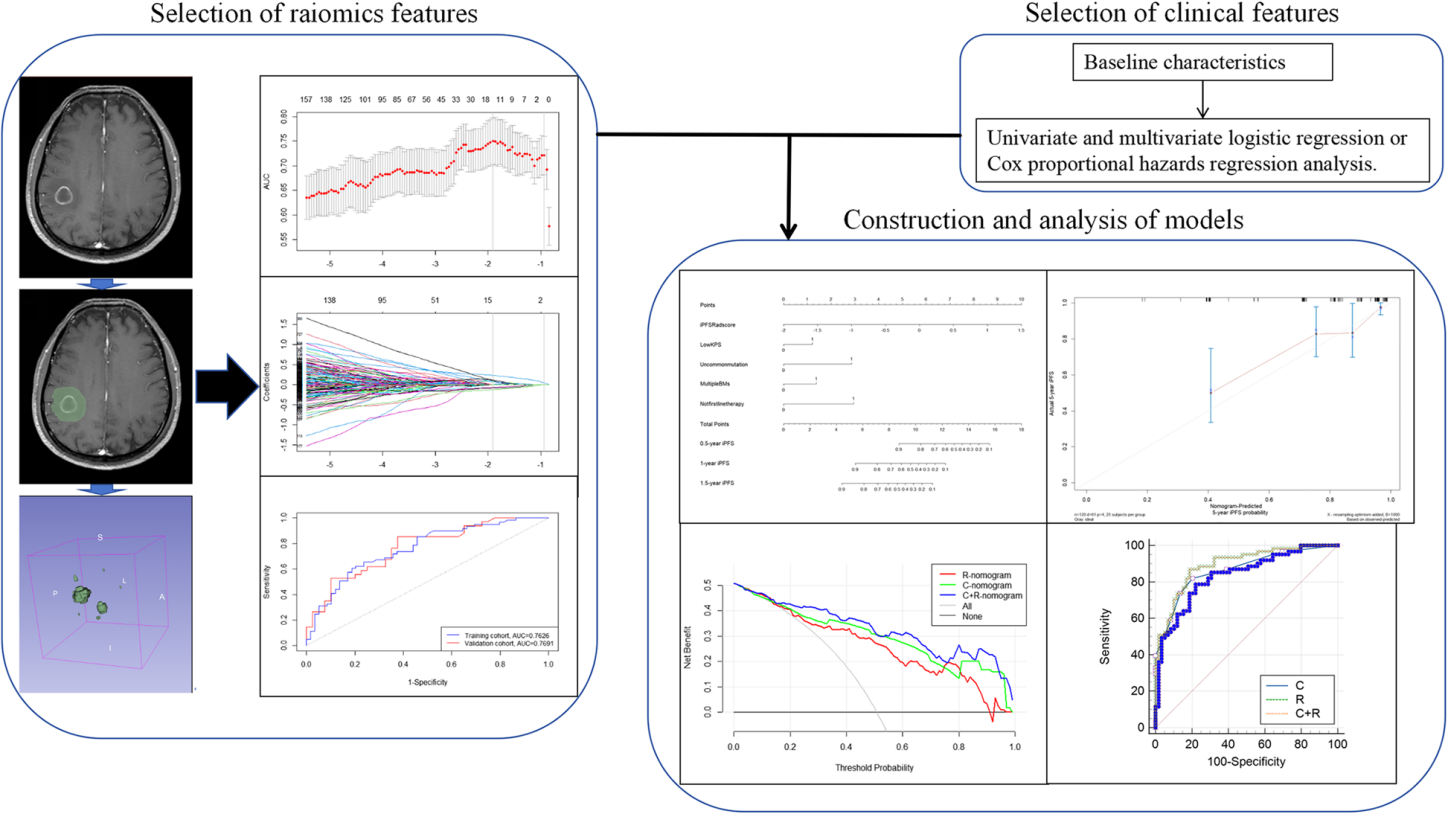
The general research process of this study

Retrospective data was collected from EGFR-mutated lung adenocarcinoma patients with brain metastasis diagnosed at Shandong Cancer Hospital between January 1, 2017, and March 1, 2023. As the study was retrospective, patient informed consent was not required and had been exempted by the institutional review board and ethics committee of Shandong Cancer Hospital. The inclusion criteria were as follows: (1) histopathologically confirmed lung adenocarcinoma through lung puncture, fiberoptic bronchoscopy or open surgery; (2) brain MRI examination performed within 3 weeks before treatment at our hospital with available MR images; (3) presence of at least one visible brain metastasis on MRI; (4) confirmation of EGFR gene mutation through amplification refractory mutation system (ARMS) or next-generation sequencing (NGS); (5) treated with third-generation EGFR-TKIs including osimertinib, almonertinib, or furmonertinib. Exclusion criteria were as follows: (1) extensive meningeal metastases accompanying brain metastases; (2) poor quality MR images hindering accurate identification of brain metastases or delineation of the region of interest (ROI); (3) negative T790M status after previous EGFR-TKI therapy; (4) combination therapy involving craniocerebral radiation therapy, surgery or other local treatments alongside third-generation EGFR-TKIs. Figure 1 illustrated the general research process for this study. The process of determining the final radiomic features mainly involved MR images acquisition and extraction and analysis of radiomics feature, while the final determination of clinical features was achieved through univariate and multivariate logistic regression analysis and cox proportional hazards regression analysis.

### MR image acquisition

All patients were positioned supine using the same equipment, a GE 3.0T superconducting MRI scanner (Discovery 750w, GE Medical, United States), for brain MRI. The brain MRI sequences included T1-weighted enhanced scan (T1 + C), T2-weighted fluid attenuated inversion recovery sequence (T2 FLAIR), diffusion-weighted imaging (DWI), T1-weighted (T1), and T2-weighted (T2) scans. During the T1 + C sequence, patients received a paramagnetic contrast agent injected into the antecubital vein at a rate of 2.2 ml/s after routine MRI and underwent examination again 5 min later. The layer spacing of brain MRI images was 3 mm. Complete MR images were retrievable from the workstation and exportable in DICOM format for ROI delineation and feature extraction.

### Extraction and analysis of radiomics feature

In this study, the brain MR image processing steps were in line with the standard flow of previous radiomics prediction models and adhered to the Image Biomarker Standardization Initiative (IBSI) [15, 16]. Initially, all MR images were preprocessed to enhance the reliability of image data analysis. The voxel size was resampled to 1 × 1 × 1 mm³ with standardized voxel spacing. Voxel intensity values were discretized using a fixed bin width of 25 HU to achieve stable intensity resolution across all images. Standardization of the image grayscale values was carried out to mitigate the impact of variation in radiomic features caused by inconsistent MR imaging parameters. Two experienced radiotherapy doctors proficient in identifying brain metastases through MRI utilized 3D-Slicer [17] (version 5.0.3, http://www.slicer.org) to delineate ROIs layer by layer on the T1 + C images of all patients in the axial plane. Additionally, adjustments were made in the sagittal and coronal planes for three-dimensional reconstruction. The delineation included the areas of brain metastases and surrounding edema. Subsequently, using the open-source Radiomics plugin within the 3D-Slicer software, radiomics features were extracted from the ROIs of all patients following preprocessing with a wavelet algorithm. All extracted radiomics features were standardized using z-scores to ensure comparability across different feature dimensions. Subsequently, brain MR images from 30 randomly selected patients underwent secondary ROI delineation and radiomics feature extraction. The radiomics features extracted in two rounds were analyzed for ICC using MATLAB R2015b (version 8.6, https://ww2.mathworks.cn/), and features with good consistency (ICC values greater than 0.8) were preliminarily identified. Then, Spearman correlation analysis was used to eliminate redundant features (|r| values between 0.8 and 1). Finally, the least absolute shrinkage and selection operator (LASSO) regression was conducted using R (version 4.2.2, http://www.r-project.org), combined with ten-fold cross-validation, to identify the optimal features. Subsequently, the radiomics signatures for short-term efficacy (SE) model and iPFS model were constructed, respectively [18, 19]. Receiver operating characteristic (ROC) curves were generated for the training and validation cohorts, and the predictive performance of the short-term efficacy and iPFS radiomics signatures was assessed based on the area under the curve (AUC) value [20].

### Calculation and processing of radiomics signature based radiomics score (rad-score)

Rad-score was calculated by multiplying the value of each feature by its corresponding coefficient and summing all the products and the constant. In this study, two types of radiomics signatures, namely the short-term efficacy (SE) signature and the intracranial progression-free survival (iPFS) signature, were developed to compute the rad-scores for each patient in the SE prediction model and the iPFS prediction model, respectively. X-tile [21] (version 3.6.1, Yale University School of Medicine, New Haven, Conn) was utilized to combine iPFS, iPFS rad-score, and progression status to calculate the optimal cut-off value for iPFS rad-scores. Subsequently, patients were categorized into high rad-score and low rad-score groups based on this cut-off value, and an analysis was performed to ascertain differences in iPFS between these two groups. In addition, logistic regression was used to analyze the correlation between the short-term efficacy rad-score and the actual short-term efficacy.

### Selection of clinical characteristics

This study considered several potential clinical factors contributing to the efficacy of third-generation EGFR-TKI, including age, sex, Karnofsky Performance Status (KPS) scores, smoking status, primary lesion location, EGFR mutation subtype, number and volume of brain metastases, location of brain metastases, brain metastasis symptoms, T and N stages according to the 8th edition lung cancer stage classification of the International Union Against Cancer (UICC) [22], presence of liver, bone, pleural, and adrenal metastases. Additionally, whether the treatment was combined with chemotherapy or anti-angiogenic therapy and whether third-generation EGFR-TKI was used as first-line therapy were also included. Univariate logistic regression analysis was employed to determine the factors associated with the short-term efficacy of the third-generation EGFR-TKI. Subsequently, multivariate logistic regression analysis was used to identify independent risk factors for short-term efficacy. Similarly, univariate Cox proportional hazard regression analysis was utilized to identify factors associated with iPFS of third-generation EGFR-TKI, followed by multivariate Cox proportional hazard regression analysis to determine independent risk factors for iPFS. Statistical significance was set at *p* < 0.05.

### Construction and validation of prediction model

Nomograms for clinical characteristics alone (C), radiomics alone (R), and the combination of clinical characteristics with radiomics (C + R) were developed to predict short-term efficacy and iPFS. The predictive performance of the nomograms was evaluated using the Harrell consistency index (C-index) [23] and the AUC values. Calibration curves were utilized to visually assess the consistency between the predicted risk of the model and the actual results [24]. Additionally, decision curve analysis (DCA) [25, 26] and Delong test were employed to compare the prediction performance of different C, R, and C + R models. Finally, we calculated the total points of each patient in the optimal predictive model, determined the cut-off values for these scores, and conducted Kaplan-Meier analysis of high-risk and low-risk patients in the overall cohort, training cohort, and validation cohort, respectively.

### Follow-up

Patients with EGFR-mutated lung adenocarcinoma and brain metastases, included in this study, underwent brain MRI and chest CT re-evaluations every 1–3 months subsequent to the initial treatment with third-generation EGFR-TKI. Short-term efficacy was determined by assessing changes in the patient’s condition at the first review following third-generation EGFR-TKI treatment compared to their condition before treatment. Systemic efficacy was assessed based on RECIST 1.1 [27] criteria, categorized as complete response (CR), partial response (PR), stable disease (SD), and progressive disease (PD). Intracranial efficacy was evaluated using RANO [28] criteria and classified into intracranial complete response (iCR), intracranial partial response (iPR), intracranial stable disease (iSD), and intracranial progressive disease (iPD). The objective response rate (ORR) denoted the proportion of patients achieving CR and PR, while the disease control rate (DCR) represented the proportion of patients with CR, PR, and SD. IPFS was defined as the duration from the initiation of third-generation EGFR-TKI therapy to either the first progression of an intracranial tumor or death due to tumor-related factors. Here, good efficacy encompassed CR and PR, while poor efficacy encompassed SD and PD. Patients surpassing the median iPFS duration were considered to have a favorable response, whereas those progressing in less time than the median iPFS were considered to have a poor response.

### Statistical analysis

In the study, IBM SPSS (version 26.0, https://www.ibm.com) was utilized to randomly divide patients into training and validation cohorts in a 5:3 ratio. Subsequently, univariate and multivariate logistic regression analyses, as well as univariate and multivariate Cox proportional regression analyses, were conducted. The two-independent sample t-test was employed for continuous variables, while the Chi-square test was applied to categorical variables. LASSO regression of the radiomics features was carried out using the glmnet package in R, and receiver operating characteristic (ROC) curves were generated. Packages such as Rms, Hmisc, lattice, survival, survminer, Formula, ggplot2, MASS and nomogramFormula in R were used to create Kaplan-Meier curves, nomograms, calibration curves, calculate the c-index and the total points. MedCalc (version 22.009, https://www.medcalc.org/) was used to plot ROC curves and perform Delong test to compare the AUC values of different nomograms. Additionally, Kaplan-Meier analysis and log-rank test was employed to assess the disparity in iPFS between high and low rad-score groups, as well as between high-risk and low-risk groups.

## Results

### Patients and clinical characteristics

A total of 194 EGFR-mutated lung adenocarcinoma patients with brain metastases receiving third-generation EGFR-TKI were enrolled in this study. The follow-up period concluded on November 10, 2023, during which intracranial progression was observed in 181 patients. The median follow-up time for the entire cohort was 23.3 months. Of the total, 120 patients were part of the training cohort, while 74 were included in the validation cohort. There were no notable differences between the training and validation cohorts concerning various factors such as age, sex, KPS, smoking status, primary lesion location, EGFR mutation subtype, number, volume, location, and symptoms of brain metastases, T stage, N stage, presence of liver metastasis, bone metastasis, pleural metastasis, adrenal metastasis, as well as whether third-generation EGFR-TKI was combined with chemotherapy or anti-angiogenic therapy, and whether third-generation EGFR-TKI was used as first-line treatment. A detailed presentation of the baseline characteristics of the patients can be found in Table 1.

**Table 1:** Baseline characteristics of EGFR-mutated lung adenocarcinoma patients with brain metastases in the training cohort and validation cohort

Clinical Characteristics	Training set(*n* = 120)	Validation set(*n* = 74)	*P* value
Gender			0.650
Male	49(40.8%)	27(36.5%)	
Female	71(59.2%)	47(63.5%)	
Age			0.462
< 60	63(52.5%)	43(58.1%)	
≥ 60	57(47.5%)	31(41.9%)	
Smoker			0.851
Yes	23(19.2%)	13(17.6%)	
No	97(80.8%)	61(82.4%)	
KPS			0.359
< 80	72(60.0%)	50(67.6%)	
≥ 80	48(40.0%)	24(32.4%)	
Location			0.293
Left lung	68(56.7%)	48(64.9%)	
Right lung	52(43.3%)	26(35.1%)	
EGFR mutation type			0.339
Common	115(95.8%)	68(91.9%)	
Uncommon	5(4.2%)	6(8.1%)	
Number of BMs			1.000
≤ 5	70(58.3%)	43(58.1%)	
> 5	50(41.7%)	31(41.9%)	
Volume of BMs			0.348
Location of BMs			0.548
Only in the hemispheres of the brain	52(43.3%)	28(37.8%)	
Exist in other location	68(56.7%)	46(62.2%)	
Symptoms of BM			0.701
Yes	20(16.7%)	14(18.9%)	
No	100(83.3%)	60(81.1%)	
T categories of TNM			0.314
= 1	35(29.2%)	16(21.6%)	
> 1	85(70.8%)	58(78.4%)	
N categories of TNM			0.296
< 3	73(60.8%)	39(52.7%)	
≥ 3	47(39.2%)	35(47.3%)	
Hepatic metastases			0.315
Yes	22(18.3%)	9(12.2%)	
No	98(81.7%)	65(87.8%)	
Bone metastases			0.765
Yes	70(58.3%)	41(55.4%)	
No	50(41.7%)	33(44.6%)	
Pleural metastases			0.255
Yes	19(15.8%)	17(23.0%)	
No	101(84.2%)	57(77.0%)	
Adrenal metastases			
Yes	14(11.7%)	7(9.5%)	
No	106(88.3%)	67(90.5%)	
Combined with chemotherapy or anti blood vessel			0.124
Yes	37(30.8%)	31(41.9%)	
No	83(69.2%)	43(58.1%)	
First-line therapy			0.655
Yes	71(59.2%)	41(55.4%)	
No	49(40.8%)	33(44.6%)	

*Abbreviation: BM *Brain metastases

### Treatment efficacy

The efficacy of the third-generation EGFR-TKI was summarized in Table 2. The assessment revealed that out of the total patients, 138 were categorized as PR, 40 as SD, and 16 as PD, resulting in an ORR of 71.1% and a DCR of 91.8%. Furthermore, 34 patients were evaluated as iCR, 106 as iPR, 31 as iSD, and 23 as iPD, leading to an iORR of 72.2% and an iDCR of 88.2%. The median iPFS was calculated at 15.67 months (7.88–20.26, interquartile range [IQR]). Based on this, 138 patients were considered to have experienced a good effect, while 56 patients were regarded as having a poor effect. Additionally, 97 patients, iPFS shorter than 15.67 months, were considered poor responses and 97 patients, iPFS longer than 15.67 months, were considered good responses.

**Table 2:** Efficacy of third-generation EGFR-TKIs

Short-term efficacy		
	PR	138(71.1%)
	SD	40(20.6%)
	PD	16(8.2%)
ORR		71.1%
DCR		91.8%
	iCR	34(17.5%)
	iPR	106(54.6%)
	iSD	31(15.9%)
	iPD	23(11.8%)
iORR		72.2%
iDCR		88.2%
miPFS	15.67months (7.88–20.26, IQR)	

*Abbreviation*: *IQR *Interquartile range

### Calculation and processing of radiomics signature based radiomics score (rad-score)

Firstly, we extracted 851 radiomics features from the ROI delineated by each patient, and then again extracted these features from a second delineation of the ROI from 30 patients. All initially extracted 851 radiomic features were presented in Supplementary Materials Table S1. Subsequently, we conducted t-tests and ICC tests on the different features extracted from these 30 patients in the two delineations, preliminarily selecting 784 radiomic features that met the criteria of *p* < 0.05 and *r* > 0.8. Finally, 16 features were selected to construct the short-term efficacy prediction model, and 13 features were selected for the iPFS prediction model using LASSO regression. These selected features were utilized to construct radiomics signatures, and the rad-scores of patients in the SE model and iPFS model were calculated. Detailed process of LASSO regression was shown in Supplementary Material Figures S1, S2 and S3 and the filtered optimal features were presented in Table 3.

**Table 3:** Features final filtered in iPFS prediction model and short-term efficacy model

Number	Radiomics features	Coefficients
iPFS		
41	originalshapeLeastAxisLength	0.0752947482352709
43	originalshapeMaximum2DDiameterColumn	0.074154613144132
44	originalshapeMaximum2DDiameterRow	0.232416684247666
269	wavelet.LHLglcmIdn	0.00291763048034297
271	wavelet.LHLglcmImc2	-0.0690107637164966
355	wavelet.LHHglcmCorrelation	0.0000419078137924003
433	wavelet.HLLfirstorderMean	0.103053204451169
457	wavelet.HLLglcmImc2	-0.0834577047415401
513	wavelet.HLLngtdmBusyness	0.0823260471375526
620	wavelet.HHLfirstorderMedian	0.0821598313036605
634	wavelet.HHLglcmCorrelation	0.00537992763534358
766	wavelet.HHHglrlmLongRunLowGrayLevelEmphasis	0.11974610886021
785	wavelet.HHHglszmSizeZoneNonUniformityNormalized	-0.120438257348373
Constant		0.0755271793882138
SE		
54	originalfirstorder90Percentile	-0.00419448768307154
62	originalfirstorderMedian	-0.00851220537927449
63	originalfirstorderMinimum	-0.00255113724154396
99	originalgldmGrayLevelNonUniformity	0.021023950190984
164	wavelet.LLHglcmAutocorrelation	-0.0301755552050827
194	wavelet.LLHgldmHighGrayLevelEmphasis	-0.0784538755713116
205	wavelet.LLHglrlmHighGrayLevelRunEmphasis	-0.123749716030615
216	wavelet.LLHglrlmShortRunHighGrayLevelEmphasis	-0.0250086674965666
244	wavelet.LHLfirstorderKurtosis	0.0451962848048691
269	wavelet.LHLglcmIdn	0.0940595066868715
346	wavelet.LHHfirstorderSkewness	-0.0133904920459787
462	wavelet.HLLglcmMCC	-0.0345285425183703
625	wavelet.HHLfirstorderSkewness	-0.150807088704218
843	wavelet.LLLgldmGrayLevelNonUniformity	0.0277809447444802
853	wavelet.LLLglrlmGrayLevelNonUniformity	0.0139511659382987
885	wavelet.LLLngtdmBusyness	0.0208305731902347
Constant		-0.634115138820642

**Fig. 2:**
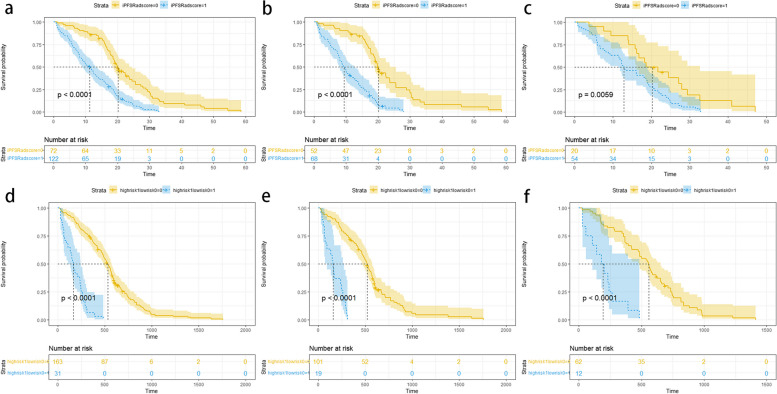
**a** Kaplan-Meier analysis of patients with high iPFS Rad-Score and low iPFS Rad-Score in the overall cohort, *p* < 0.0001. **b** Kaplan-Meier analysis of patients with high iPFS Rad-Score and low iPFS Rad-Score in the training cohort. *p* < 0.0001. **c** Kaplan-Meier analysis of patients with high iPFS Rad-Score and low iPFS Rad-Score in the validation cohort, *p* = 0.0059. **d** Kaplan-Meier survival analysis of high-risk and low-risk patients in the overall cohort, *p* < 0.0001. **e** Kaplan-Meier survival analysis of high-risk and low-risk patients in the training cohort, *p* < 0.0001. **f** Kaplan-Meier survival analysis of high-risk and low-risk patients in the validation cohort, *p* < 0.0001

In the SE prediction model, logistic regression was employed to examine the association between short-term efficacy and the SE rad-score. In the overall cohort, a significant correlation was observed (*p* < 0.001, odds ratio [OR] = 24.871 [5.712, 108.296]). Similarly, in the training cohort, a significant correlation was found (*p* < 0.001, OR = 97.744 [10.758, 888.047]). In the validation cohort, a significant correlation was also present (*p* = 0.045, OR = 8.131 [1.044, 63.303]). These findings suggest that the short-term efficacy of third-generation EGFR-TKI was strongly associated with the SE rad-score. Regarding the iPFS prediction model, the cut-off value for the iPFS rad-score was determined as -0.1. Consequently, patients with a rad-score greater than − 0.1 were classified into the high-risk group, while those with a rad-score less than − 0.1 were assigned to the low-risk group. Kaplan-Meier survival analysis demonstrated a significant correlation between iPFS and the iPFS rad-score. Detailed Kaplan-Meier curves and log-rank test were shown in Fig. 2a, b and c.

### Relevant factors in iPFS and short-term efficacy models

Univariate regression analysis revealed that several factors were associated with poorer iPFS. These factors included age ≥ 60, KPS < 80, uncommon EGFR mutation, the presence of more than 5 brain metastases, an increase in the volume of brain metastases, brain metastases not limited to the cerebral hemisphere, and receiving non-first-line treatment with third-generation EGFR-TKI. Additionally, KPS < 80, uncommon EGFR mutations, and non-first-line therapy with third-generation EGFR-TKI were found to be linked to poor short-term outcomes. Further multivariate regression analysis was conducted to determine independent risk factors for poor iPFS. The analysis identified *LowKPS* (Binary variable, KPS < 80), *Uncommonmutation (*Binary variable, EGFR uncommon mutations*)*, *MultipleBMs* (Binary variable, BM number more than 5), and *Notfirstlinetherapy* (Binary variable, EGFR-TKI was not used as a first-line treatment) as independent risk factors. Similarly, *LowKPS*, *Uncommonmutation*, and *Notfirstlinetherapy* were identified as independent risk factors for poor short-term efficacy. Detailed univariate and multifactor logistic regression analysis results could be found in Table 4, while univariate and multifactor Cox regression analysis results were presented in Table 5.

**Table 4:** Univariable and multivariable logistic regression analysis of risk factors potentially associated with short-term efficacy in EGFR-mutated lung adenocarcinoma patients with brain metastases

Characteristics	Univariable analysis *p* value	Multivariable analysis			
		*P* value	Exp(B)	95% CI	
Gender (Male vs. Female)	0.733				
Age (≥ 60 vs.<60)	0.185				
Smoker (Yes vs.No)	0.291				
KPS (< 80 vs.≥80)	0.022	0.016	0.402	0.192	0.843
Location (Left lung vs.Right)	0.956				
EGFR mutation type (common vs. uncommon)	0.027	0.029	5.155	1.184	22.455
Number of BMs (≤ 5 vs.>5)	0.108				
Volume of BMs	0.216				
Location of BMs (Only in the hemispheres of the brain vs. Exists in other location)	0.438				
Symptoms of BM (Yes vs.No)	0.084				
T categories of TNM (1 vs.>1)	0.825				
N categories of TNM (< 3 vs.≥3)	0.287				
Hepatic metastases (Yes vs.No)	0.750				
Bone metastases (Yes vs.No)	0.158				
Pleural metastasis (Yes vs.No)	0.357				
Adrenal metastasis (Yes vs.No)	0.052				
Combined with chemotherapy or antiangiogenic therapy (Yes vs.No)	0.927				
First-line therapy (Yes vs.No)	0.000	0.000	0.147	0.073	0.295

*Abbreviations*: *Exp(B) *odds ratio, *CI *Confidence interval, *BM *Brain metastases

**Table 5:** Univariable and multivariable cox proportional hazard regression analysis of risk factors potentially associated with iPFS in EGFR-mutated lung adenocarcinoma patients with brain metastases

Characteristics	Univariable analysis *p* value	Multivariable analysis			
		*P* value	Exp(B)	95% CI	
Gender (Male vs. Female)	0.080				
Age (≥ 60 vs.<60)	0.027	0.134	1.394	0.903	2.153
Smoker (Yes vs.No)	0.388				
KPS (< 80 vs.≥80)	0.008	0.008	1.933	1.190	3.139
Location (Left lung vs.Right)	0.889				
EGFR mutation type (Common vs. Uncommon)	0.005	0.005	0.328	0.150	0.716
Number of BMs (≤ 5 vs.>5)	0.000	0.000	0.163	0.098	0.272
Volume of BMs	0.008	0.182	1.000	1.000	1.000
Location of BMs (Only in the hemispheres of the brain vs. Exist in other location)	0.002	0.829	1.058	0.636	1.758
Symptoms of BM (Yes vs.No)	0.455				
T categories of TNM (1 vs.>1)	0.133				
N categories of TNM (< 3 vs.≥3)	0.725				
Hepatic metastases (Yes vs.No)	0.124				
Bone metastases (Yes vs.No)	0.277				
Pleural metastasis (Yes vs.No)	0.496				
Adrenal metastasis (Yes vs.No)	0.316				
Combined with chemotherapy or antiangiogenic therapy (Yes vs.No)	0.608				
First-line therapy (Yes vs.No)	0.000	0.000	2.880	1.869	4.437

*Abbreviations*: *Exp(B) *odds ratio, *CI *Confidence interval, *BM *Brain metastases

### Performance assessment of nomograms

**Fig. 3:**
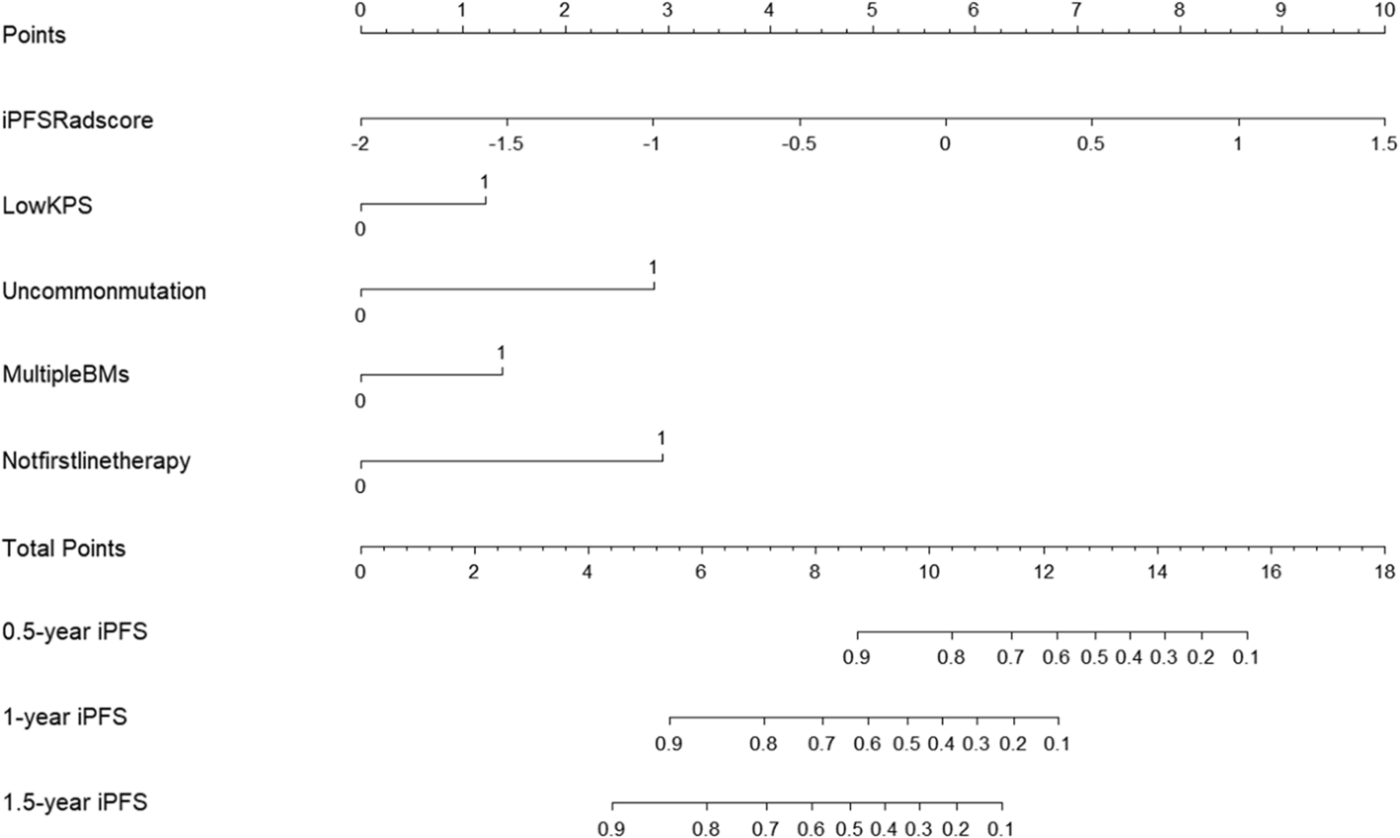
Clinical plus radiomics nomogram of iPFS model

**Fig. 4:**
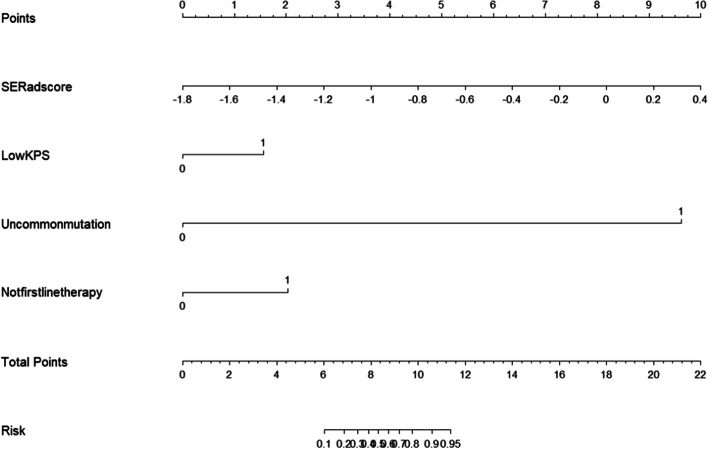
Clinical plus radiomics nomogram of short-term efficacy model

**Fig. 5:**
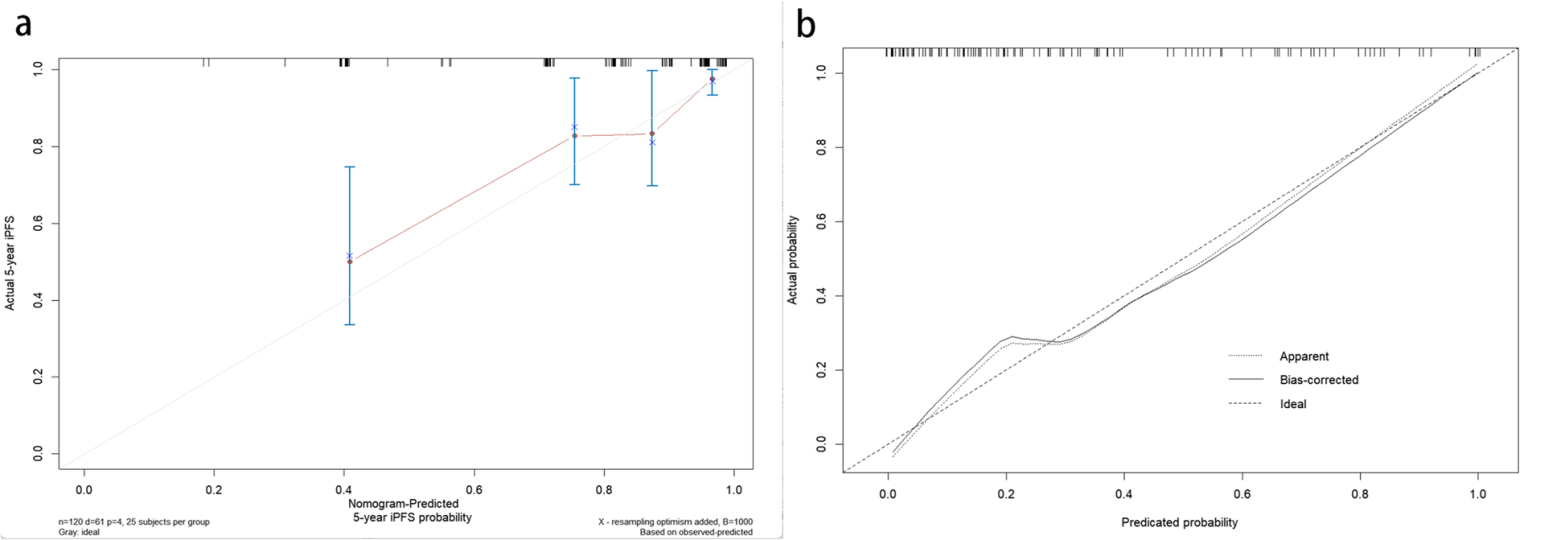
**a** Calibration curve of iPFS clinical plus radiomics nomogram. **b **Calibration curve of short-term efficacy clinical plus radiomics nomogram

**Fig. 6:**
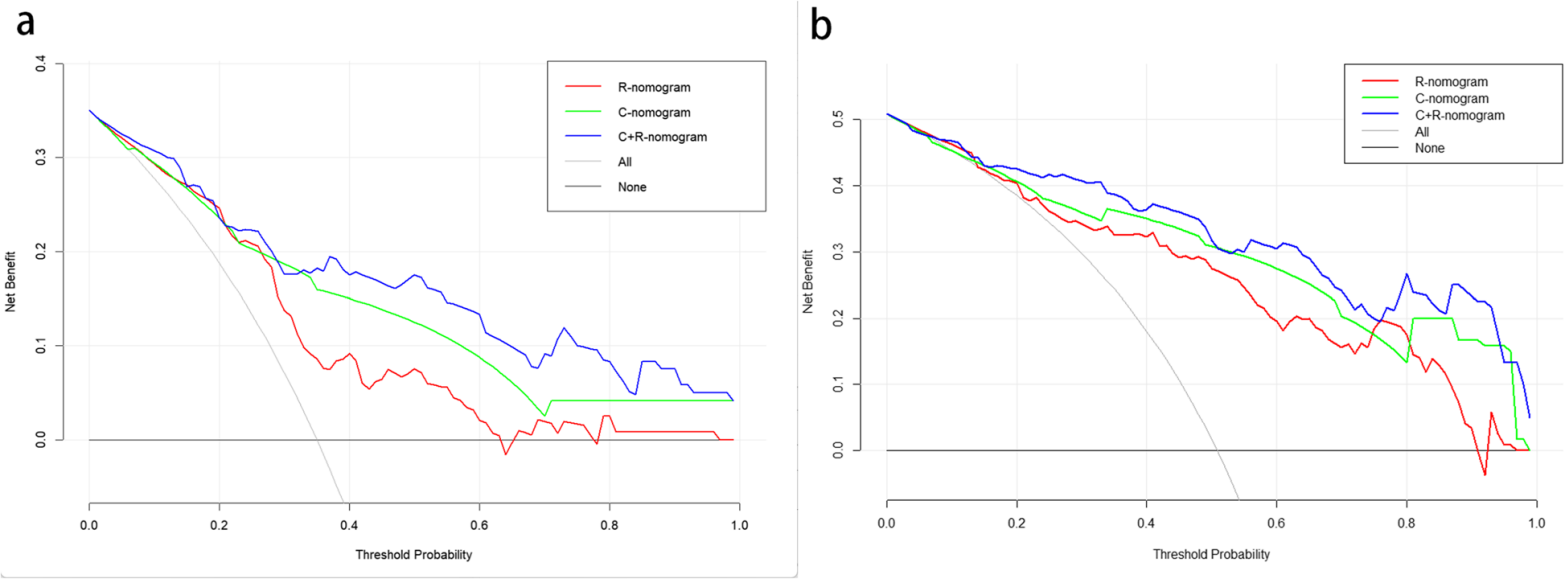
**a** DCA curves of short-term efficacy nomograms. **b** DCA curves of iPFS nomograms. R, DCA curve of radiomics nomogram; C, DCA curve of clinical nomogram; C + R, DCA curve of clinical plus radiomics nomogram

**Fig. 7:**
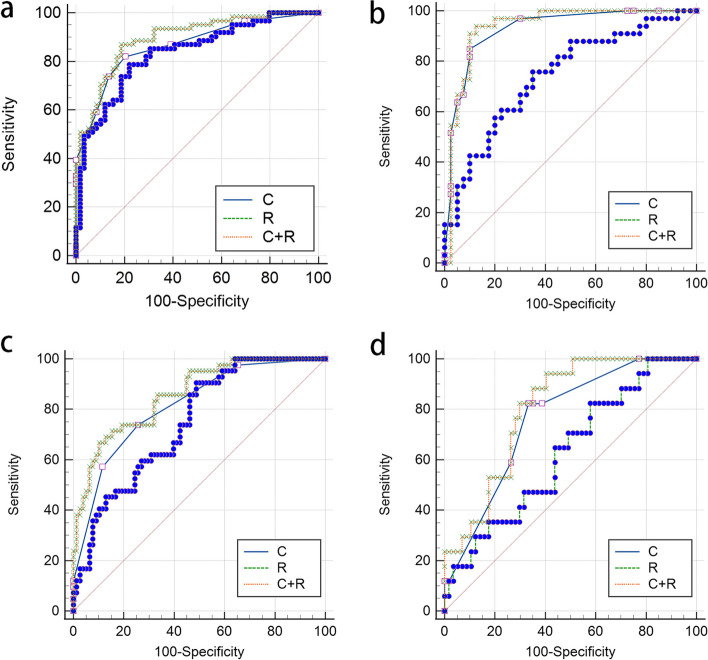
ROC curves for the training cohort (**a**) and validation cohort (**b**) of the iPFS model, as well as the training cohort (**c**) and validation cohort (**d**) of the SE model

In the short-term efficacy prediction model, the C-indexes of C + R, C and R nomograms in the training cohort were 0.867 (0.835-0.900, 95%CI), 0.815 (0.77–0.853, 95%CI), and 0.747 (0.703–0.791, 95%CI), respectively. In the validation cohort, the C-indexes of C + R, C and R nomograms were 0.803 (0.753–0.854, 95%CI), 0.762 (0.706–0.818, 95%CI) and 0.628 (0.555–0.702, 95%CI), respectively. For the iPFS prediction model, the C-indexes of C + R, C and R nomograms in the training cohort were 0.901 (0.874–0.929, 95%CI), 0.863 (0.831–0.896, 95%CI) and 0.835 (0.799–0.872, 95%CI), respectively. In the validation cohort, the C-indexes of C + R, C and R nomograms were 0.753 (0.713–0.793, 95%CI), 0.803 (0.77–0.836, 95%CI) and 0.654 (0.610–0.698, 95%CI), respectively. The combined C + R nomograms constructed in the iPFS model and short-term efficacy model were shown in Figs. 3 and 4, respectively. And the remaining single C or R nomograms could be found in Supplementary Material Figure S4. The calibration curves in Figs. 5 and 6 and Supplementary Material Figure S5 indicated strong agreement between the predicted probability and actual observations of the C + R models developed in this study. Additionally, the DCA illustrated in Fig. 5 highlighted that the combined C + R models outperformed the single C or R models in terms of prediction performance. The ROC curves and AUC values for different nomograms were displayed in Fig. 7; Table 6. Finally, the cut-off value for the total points of patients in the iPFS predictive model was 12.0, and Kaplan-Meier analysis showed a significant difference in the iPFS between high-risk and low-risk patients in the overall cohort, training cohort, and validation cohort, with all *p*-values < 0.0001 (Fig. 2d, e and f).

**Table 6:** Comparison of AUC values between different nomograms

		Training cohort				Validation cohort			
		AUC(95% CI)	*P* value			AUC(95% CI)	*P* value		
			C	R	C + R		C	R	C + R
iPFS	C	0.866(0.791–0.921)	-	0.527	0.502	0.928(0.843–0.976)	-	0.005	0.030
	R	0.835(0.756–0.896)	-	-	0.196	0.744(0.628–0.839)	-	-	0.003
	C + R	0.895(0.825–0.943)	-	-	-	0.936(0.854–0.980)	-	-	-
SE	C	0.815(0.734–0.880)	-	0.245	0.402	0.762(0.648–0.853)	-	0.157	0.587
	R	0.747(0.660–0.822)	-	-	0.048	0.628(0.508–0.738)	-	-	0.054
	C + R	0.858(0.783–0.915)	-	-	-	0.804(0.695–0.887)	-	-	-

*Abbreviations*: *CI *Confidence interval, *C *Clinical nomogram, *R *Radiomics nomogram, *C + R *Clinical and radiomics combined nomogram

## Discussion

In this study, we had successfully developed and validated radiomics nomograms based on clinical characteristics and MRI to predict the short-term efficacy and iPFS of third-generation EGFR-TKIs, including osimertinib, almonertinib and furmonertinib, for lung adenocarcinoma patients with EGFR-mutated brain metastases. There was good consistency between predicted risks and actual outcomes. We compared C, R, and C + R nomograms, confirming that the combined C + R nomogram outperforms the individual C or R nomograms. Predictive factors in the short-term efficacy nomogram include SE rad-score, low KPS (KPS < 80), uncommon EGFR mutations, and not first-line treatment with third-generation EGFR-TKIs. Predictive factors in the iPFS nomogram include iPFS rad-score, low KPS (KPS < 80), uncommon EGFR mutations, multiple brain metastases, and not first-line treatment with third-generation EGFR-TKIs.

This study ultimately identified 16 radiomic features associated with third-generation EGFR-TKIs short-term efficacy and 13 radiomic features related to iPFS, which were used to construct radiomics signatures and calculate SE rad-score and iPFS rad-score for each patient. The optimal radiomics features associated with short-term efficacy included 6 first-order features, 3 Gy level dependence matrix (GLDM) features, 3 Gy-level co-occurrence matrix (GLCM) features, 3 Gy-level run length matrix (GLRLM) features, and 1 non-uniformity of gray tones and distance matrix (NGTDM) feature. The optimal radiomics features related to iPFS included 5 GLCM features, 3 shape features, 2 first-order features, 1 NGTDM feature, 1 GLRLM feature, and 1 Gy-level size zone matrix (GLSZM) feature. Among them, wavelet.LHL_glcm_Idn was a feature obtained by combining wavelet transform with gray level co-occurrence matrix (GLCM) in a specific subband (LHL) and performing inverse difference normalization (IDN). This feature was included in both the selected features of the short-term efficacy model and the iPFS model. We compared previous radiomics studies on EGFR-mutated NSCLC brain metastases [29–31], and although the selected features varied due to different research directions and screening processes, there were still a few features that overlapped with our study, indicating their general applicability in EGFR-mutated NSCLC brain metastasis.

KPS reflected the physical activity and functional status of patients, and higher KPS in cancer patients were generally associated with better overall physical condition and higher tolerance. While low KPS in patients affected drug absorption, metabolism, and excretion, as well as the immune function and compliance of the body. Previous studies had confirmed that the KPS could be used to predict post-discharge mortality in patients with liver cirrhosis [32], prognosis in stage I non-small cell lung cancer [33], and survival in advanced pancreatic cancer patients [34]. Patients with lower KPS scores had a higher predictive risk in the nomograms of this study, and the use of third-generation EGFR-TKIs for anti-tumor treatment should be conducted under the premise of improving the overall condition of patients as much as possible.

In this study, uncommon EGFR mutations encompassed 20 exon insertion mutations, G718X, S768I, and L861Q. Similar to the research conducted by Wu et al. [35], which found poor treatment responses of first-generation EGFR-TKIs to uncommon mutations, our study also identified uncommon EGFR mutations as independent risk factors for the efficacy of third-generation EGFR-TKIs. Additionally, Yang et al. [36, 37] confirmed that afatinib exhibited a poor response to exon 20 insertion mutations but showed good responses to other uncommon mutations such as G719X, S768I, and L861Q. It should be noted that while third-generation EGFR-TKIs have shown promising results, they cannot completely replace previous medications. Future targeted drug development should consider adjusting for uncommon mutations. Within the nomograms constructed in our study, uncommon mutations were associated with a higher predictive risk. If feasible, patients could benefit from receiving third-generation EGFR-TKI treatment after modifying their uncommon EGFR mutation status.

EGFR-mutated lung adenocarcinoma patients with brain metastases typically experience shorter survival. A multicenter clinical trial [38] demonstrated that in EGFR-mutated NSCLC patients with brain metastases, first-line gefitinib treatment resulted in a median iPFS of 9.1 months and a median overall survival (OS) of 28.9 months. However, after multiple disease progressions, patients did not significantly benefit from third-generation EGFR-TKI therapy due to compromised overall health. Furthermore, a study by Bai H et al. [39] suggested that chemotherapy might affect the EGFR mutation status of NSCLC patients, as tumors can display heterogeneity in their mutational profiles. In comparison to other treatments, first-line therapy with third-generation EGFR-TKIs had fewer adverse reactions and could help maintain better overall health even after disease progression, enabling further treatment options. For patients already receiving non-first-line therapy, the benefit of treatment with third-generation EGFR-TKIs alone was not substantial. Therefore, combination therapy should be considered to improve patient outcomes.

The number of brain metastases in patients with EGFR-mutated lung adenocarcinoma could serve as a reflection of the tumor burden within the brain. However, visible metastases on MRI scans were often limited, making it challenging to assess the full extent of brain involvement. Higher numbers of brain metastases indicate a greater tumor burden, making it more difficult to achieve control over the disease. Chang et al. [40] confirmed that patients with a higher number of brain lesions were more likely to experience disease progression. For EGFR-mutated lung adenocarcinoma patients with a higher number of brain metastases, the timely addition of cranial radiotherapy has been shown to benefit these patients [41]. Regarding the volume of brain metastases, Baschnagel et al. [42] demonstrated that tumor volume independently predicts the efficacy of Gamma Knife surgery in treating brain metastases. Although this current study did not consider the increase in brain metastasis volume as an independent risk factor, it did confirm through univariate Cox regression analysis that the volume of brain metastases is associated with iPFS.

There were also some limitations in this study. Firstly, it was a single-center study, so the predictive performance of the model needed further validation in different regions and among different ethnic groups. Secondly, being a retrospective study, it inevitably suffered from selection bias and confounding factors. Furthermore, despite efforts to mitigate these issues, the feature selection process involved multiple steps and could be subject to subjectivity and human bias. Finally, the differences in the efficacy of the three different third-generation EGFR-TKIs as well as the differences in the proportion of patients receiving treatment with each drug may impact the study results.

## Conclusions

The third-generation EGFR-TKIs have significantly improved the efficacy and prognosis of patients with EGFR-mutated lung adenocarcinoma and brain metastases. The predictive potential of radiomics is indeed real, and combining clinical characteristics with radiomic nomograms based on MRI can be used for short-term efficacy and iPFS prediction in these patients. It serves as a non-invasive predictive tool, aiding physicians and patients in better understanding prognosis risks and allowing for personalized adjustments to patient treatment plans in a timely manner.

### Supplementary Information

**Supplementary Material 1.**

## Data Availability

No datasets were generated or analysed during the current study.

## Abbreviations

EGFR-mutated: Epidermal growth factor receptor gene mutated
EGFR-TKI: Epidermal growth factor receptor tyrosine kinase inhibitor
MRI: Magnetic resonance imaging
iPFS: Intracranial progression free survival
LASSO: Least absolute shrinkage and selection operator
C: Single clinical
R: Single radiomics
C + R: Combined clinical and radiomics
C-index: Harrell’s concordance index
DCA: Decision curve analysis
ORR: Overall response rate
DCR: Disease control rate
IQR: Interquartile range
NSCLC: Non-small cell lung cancer
OS: Overall survival
ROC: Receiver operating characteristic
AUC: Area under the curve
SE: Short-term efficacy
