# Supplementary material for: Clinical characteristics and MRI based radiomics nomograms can predict iPFS and short-term efficacy of third-generation EGFR-TKI in EGFR-mutated lung adenocarcinoma with brain metastases
Source: BMC Cancer. 2024 Mar 21;24:362. doi: 10.1186/s12885-024-12121-z (PMC10956298; doi:10.1186/s12885-024-12121-z)

**Supplementary Material Table S1.** The 851 initially extracted radiomic features from the ROI.

| **Number** | **originalshape** | **original** | **wavelet-LLH** | **wavelet-LHL** | **wavelet-LHH** | **wavelet-HLL** | **wavelet-HLH** | **wavelet-HHL** | **wavelet-HHH** | **wavelet-LLL** |
| --- | --- | --- | --- | --- | --- | --- | --- | --- | --- | --- |
| 1 | originalshapeElongation | originalfirstorder10Percentile | wavelet-LLHfirstorder10Percentile | wavelet-LHLfirstorder10Percentile | wavelet-LHHfirstorder10Percentile | wavelet-HLLfirstorder10Percentile | wavelet-HLHfirstorder10Percentile | wavelet-HHLfirstorder10Percentile | wavelet-HHHfirstorder10Percentile | wavelet-LLLfirstorder10Percentile |
| 2 | originalshapeFlatness | originalfirstorder90Percentile | wavelet-LLHfirstorder90Percentile | wavelet-LHLfirstorder90Percentile | wavelet-LHHfirstorder90Percentile | wavelet-HLLfirstorder90Percentile | wavelet-HLHfirstorder90Percentile | wavelet-HHLfirstorder90Percentile | wavelet-HHHfirstorder90Percentile | wavelet-LLLfirstorder90Percentile |
| 3 | originalshapeLeastAxisLength | originalfirstorderEnergy | wavelet-LLHfirstorderEnergy | wavelet-LHLfirstorderEnergy | wavelet-LHHfirstorderEnergy | wavelet-HLLfirstorderEnergy | wavelet-HLHfirstorderEnergy | wavelet-HHLfirstorderEnergy | wavelet-HHHfirstorderEnergy | wavelet-LLLfirstorderEnergy |
| 4 | originalshapeMajorAxisLength | originalfirstorderEntropy | wavelet-LLHfirstorderEntropy | wavelet-LHLfirstorderEntropy | wavelet-LHHfirstorderEntropy | wavelet-HLLfirstorderEntropy | wavelet-HLHfirstorderEntropy | wavelet-HHLfirstorderEntropy | wavelet-HHHfirstorderEntropy | wavelet-LLLfirstorderEntropy |
| 5 | originalshapeMaximum2DDiameterColumn | originalfirstorderInterquartileRange | wavelet-LLHfirstorderInterquartileRange | wavelet-LHLfirstorderInterquartileRange | wavelet-LHHfirstorderInterquartileRange | wavelet-HLLfirstorderInterquartileRange | wavelet-HLHfirstorderInterquartileRange | wavelet-HHLfirstorderInterquartileRange | wavelet-HHHfirstorderInterquartileRange | wavelet-LLLfirstorderInterquartileRange |
| 6 | originalshapeMaximum2DDiameterRow | originalfirstorderKurtosis | wavelet-LLHfirstorderKurtosis | wavelet-LHLfirstorderKurtosis | wavelet-LHHfirstorderKurtosis | wavelet-HLLfirstorderKurtosis | wavelet-HLHfirstorderKurtosis | wavelet-HHLfirstorderKurtosis | wavelet-HHHfirstorderKurtosis | wavelet-LLLfirstorderKurtosis |
| 7 | originalshapeMaximum2DDiameterSlice | originalfirstorderMaximum | wavelet-LLHfirstorderMaximum | wavelet-LHLfirstorderMaximum | wavelet-LHHfirstorderMaximum | wavelet-HLLfirstorderMaximum | wavelet-HLHfirstorderMaximum | wavelet-HHLfirstorderMaximum | wavelet-HHHfirstorderMaximum | wavelet-LLLfirstorderMaximum |
| 8 | originalshapeMaximum3DDiameter | originalfirstorderMeanAbsoluteDeviation | wavelet-LLHfirstorderMeanAbsoluteDeviation | wavelet-LHLfirstorderMeanAbsoluteDeviation | wavelet-LHHfirstorderMeanAbsoluteDeviation | wavelet-HLLfirstorderMeanAbsoluteDeviation | wavelet-HLHfirstorderMeanAbsoluteDeviation | wavelet-HHLfirstorderMeanAbsoluteDeviation | wavelet-HHHfirstorderMeanAbsoluteDeviation | wavelet-LLLfirstorderMeanAbsoluteDeviation |
| 9 | originalshapeMeshVolume | originalfirstorderMean | wavelet-LLHfirstorderMean | wavelet-LHLfirstorderMean | wavelet-LHHfirstorderMean | wavelet-HLLfirstorderMean | wavelet-HLHfirstorderMean | wavelet-HHLfirstorderMean | wavelet-HHHfirstorderMean | wavelet-LLLfirstorderMean |
| 10 | originalshapeMinorAxisLength | originalfirstorderMedian | wavelet-LLHfirstorderMedian | wavelet-LHLfirstorderMedian | wavelet-LHHfirstorderMedian | wavelet-HLLfirstorderMedian | wavelet-HLHfirstorderMedian | wavelet-HHLfirstorderMedian | wavelet-HHHfirstorderMedian | wavelet-LLLfirstorderMedian |
| 11 | originalshapeSphericity | originalfirstorderMinimum | wavelet-LLHfirstorderMinimum | wavelet-LHLfirstorderMinimum | wavelet-LHHfirstorderMinimum | wavelet-HLLfirstorderMinimum | wavelet-HLHfirstorderMinimum | wavelet-HHLfirstorderMinimum | wavelet-HHHfirstorderMinimum | wavelet-LLLfirstorderMinimum |
| 12 | originalshapeSurfaceArea | originalfirstorderRange | wavelet-LLHfirstorderRange | wavelet-LHLfirstorderRange | wavelet-LHHfirstorderRange | wavelet-HLLfirstorderRange | wavelet-HLHfirstorderRange | wavelet-HHLfirstorderRange | wavelet-HHHfirstorderRange | wavelet-LLLfirstorderRange |
| 13 | originalshapeSurfaceVolumeRatio | originalfirstorderRobustMeanAbsoluteDeviation | wavelet-LLHfirstorderRobustMeanAbsoluteDeviation | wavelet-LHLfirstorderRobustMeanAbsoluteDeviation | wavelet-LHHfirstorderRobustMeanAbsoluteDeviation | wavelet-HLLfirstorderRobustMeanAbsoluteDeviation | wavelet-HLHfirstorderRobustMeanAbsoluteDeviation | wavelet-HHLfirstorderRobustMeanAbsoluteDeviation | wavelet-HHHfirstorderRobustMeanAbsoluteDeviation | wavelet-LLLfirstorderRobustMeanAbsoluteDeviation |
| 14 | originalshapeVoxelVolume | originalfirstorderRootMeanSquared | wavelet-LLHfirstorderRootMeanSquared | wavelet-LHLfirstorderRootMeanSquared | wavelet-LHHfirstorderRootMeanSquared | wavelet-HLLfirstorderRootMeanSquared | wavelet-HLHfirstorderRootMeanSquared | wavelet-HHLfirstorderRootMeanSquared | wavelet-HHHfirstorderRootMeanSquared | wavelet-LLLfirstorderRootMeanSquared |
| 15 |  | originalfirstorderSkewness | wavelet-LLHfirstorderSkewness | wavelet-LHLfirstorderSkewness | wavelet-LHHfirstorderSkewness | wavelet-HLLfirstorderSkewness | wavelet-HLHfirstorderSkewness | wavelet-HHLfirstorderSkewness | wavelet-HHHfirstorderSkewness | wavelet-LLLfirstorderSkewness |
| 16 |  | originalfirstorderTotalEnergy | wavelet-LLHfirstorderTotalEnergy | wavelet-LHLfirstorderTotalEnergy | wavelet-LHHfirstorderTotalEnergy | wavelet-HLLfirstorderTotalEnergy | wavelet-HLHfirstorderTotalEnergy | wavelet-HHLfirstorderTotalEnergy | wavelet-HHHfirstorderTotalEnergy | wavelet-LLLfirstorderTotalEnergy |
| 17 |  | originalfirstorderUniformity | wavelet-LLHfirstorderUniformity | wavelet-LHLfirstorderUniformity | wavelet-LHHfirstorderUniformity | wavelet-HLLfirstorderUniformity | wavelet-HLHfirstorderUniformity | wavelet-HHLfirstorderUniformity | wavelet-HHHfirstorderUniformity | wavelet-LLLfirstorderUniformity |
| 18 |  | originalfirstorderVariance | wavelet-LLHfirstorderVariance | wavelet-LHLfirstorderVariance | wavelet-LHHfirstorderVariance | wavelet-HLLfirstorderVariance | wavelet-HLHfirstorderVariance | wavelet-HHLfirstorderVariance | wavelet-HHHfirstorderVariance | wavelet-LLLfirstorderVariance |
| 19 |  | originalglcmAutocorrelation | wavelet-LLHglcmAutocorrelation | wavelet-LHLglcmAutocorrelation | wavelet-LHHglcmAutocorrelation | wavelet-HLLglcmAutocorrelation | wavelet-HLHglcmAutocorrelation | wavelet-HHLglcmAutocorrelation | wavelet-HHHglcmAutocorrelation | wavelet-LLLglcmAutocorrelation |
| 20 |  | originalglcmClusterProminence | wavelet-LLHglcmClusterProminence | wavelet-LHLglcmClusterProminence | wavelet-LHHglcmClusterProminence | wavelet-HLLglcmClusterProminence | wavelet-HLHglcmClusterProminence | wavelet-HHLglcmClusterProminence | wavelet-HHHglcmClusterProminence | wavelet-LLLglcmClusterProminence |
| 21 |  | originalglcmClusterShade | wavelet-LLHglcmClusterShade | wavelet-LHLglcmClusterShade | wavelet-LHHglcmClusterShade | wavelet-HLLglcmClusterShade | wavelet-HLHglcmClusterShade | wavelet-HHLglcmClusterShade | wavelet-HHHglcmClusterShade | wavelet-LLLglcmClusterShade |
| 22 |  | originalglcmClusterTendency | wavelet-LLHglcmClusterTendency | wavelet-LHLglcmClusterTendency | wavelet-LHHglcmClusterTendency | wavelet-HLLglcmClusterTendency | wavelet-HLHglcmClusterTendency | wavelet-HHLglcmClusterTendency | wavelet-HHHglcmClusterTendency | wavelet-LLLglcmClusterTendency |
| 23 |  | originalglcmContrast | wavelet-LLHglcmContrast | wavelet-LHLglcmContrast | wavelet-LHHglcmContrast | wavelet-HLLglcmContrast | wavelet-HLHglcmContrast | wavelet-HHLglcmContrast | wavelet-HHHglcmContrast | wavelet-LLLglcmContrast |
| 24 |  | originalglcmCorrelation | wavelet-LLHglcmCorrelation | wavelet-LHLglcmCorrelation | wavelet-LHHglcmCorrelation | wavelet-HLLglcmCorrelation | wavelet-HLHglcmCorrelation | wavelet-HHLglcmCorrelation | wavelet-HHHglcmCorrelation | wavelet-LLLglcmCorrelation |
| 25 |  | originalglcmDifferenceAverage | wavelet-LLHglcmDifferenceAverage | wavelet-LHLglcmDifferenceAverage | wavelet-LHHglcmDifferenceAverage | wavelet-HLLglcmDifferenceAverage | wavelet-HLHglcmDifferenceAverage | wavelet-HHLglcmDifferenceAverage | wavelet-HHHglcmDifferenceAverage | wavelet-LLLglcmDifferenceAverage |
| 26 |  | originalglcmDifferenceEntropy | wavelet-LLHglcmDifferenceEntropy | wavelet-LHLglcmDifferenceEntropy | wavelet-LHHglcmDifferenceEntropy | wavelet-HLLglcmDifferenceEntropy | wavelet-HLHglcmDifferenceEntropy | wavelet-HHLglcmDifferenceEntropy | wavelet-HHHglcmDifferenceEntropy | wavelet-LLLglcmDifferenceEntropy |
| 27 |  | originalglcmDifferenceVariance | wavelet-LLHglcmDifferenceVariance | wavelet-LHLglcmDifferenceVariance | wavelet-LHHglcmDifferenceVariance | wavelet-HLLglcmDifferenceVariance | wavelet-HLHglcmDifferenceVariance | wavelet-HHLglcmDifferenceVariance | wavelet-HHHglcmDifferenceVariance | wavelet-LLLglcmDifferenceVariance |
| 28 |  | originalglcmId | wavelet-LLHglcmId | wavelet-LHLglcmId | wavelet-LHHglcmId | wavelet-HLLglcmId | wavelet-HLHglcmId | wavelet-HHLglcmId | wavelet-HHHglcmId | wavelet-LLLglcmId |
| 29 |  | originalglcmIdm | wavelet-LLHglcmIdm | wavelet-LHLglcmIdm | wavelet-LHHglcmIdm | wavelet-HLLglcmIdm | wavelet-HLHglcmIdm | wavelet-HHLglcmIdm | wavelet-HHHglcmIdm | wavelet-LLLglcmIdm |
| 30 |  | originalglcmIdmn | wavelet-LLHglcmIdmn | wavelet-LHLglcmIdmn | wavelet-LHHglcmIdmn | wavelet-HLLglcmIdmn | wavelet-HLHglcmIdmn | wavelet-HHLglcmIdmn | wavelet-HHHglcmIdmn | wavelet-LLLglcmIdmn |
| 31 |  | originalglcmIdn | wavelet-LLHglcmIdn | wavelet-LHLglcmIdn | wavelet-LHHglcmIdn | wavelet-HLLglcmIdn | wavelet-HLHglcmIdn | wavelet-HHLglcmIdn | wavelet-HHHglcmIdn | wavelet-LLLglcmIdn |
| 32 |  | originalglcmImc1 | wavelet-LLHglcmImc1 | wavelet-LHLglcmImc1 | wavelet-LHHglcmImc1 | wavelet-HLLglcmImc1 | wavelet-HLHglcmImc1 | wavelet-HHLglcmImc1 | wavelet-HHHglcmImc1 | wavelet-LLLglcmImc1 |
| 33 |  | originalglcmImc2 | wavelet-LLHglcmImc2 | wavelet-LHLglcmImc2 | wavelet-LHHglcmImc2 | wavelet-HLLglcmImc2 | wavelet-HLHglcmImc2 | wavelet-HHLglcmImc2 | wavelet-HHHglcmImc2 | wavelet-LLLglcmImc2 |
| 34 |  | originalglcmInverseVariance | wavelet-LLHglcmInverseVariance | wavelet-LHLglcmInverseVariance | wavelet-LHHglcmInverseVariance | wavelet-HLLglcmInverseVariance | wavelet-HLHglcmInverseVariance | wavelet-HHLglcmInverseVariance | wavelet-HHHglcmInverseVariance | wavelet-LLLglcmInverseVariance |
| 35 |  | originalglcmJointAverage | wavelet-LLHglcmJointAverage | wavelet-LHLglcmJointAverage | wavelet-LHHglcmJointAverage | wavelet-HLLglcmJointAverage | wavelet-HLHglcmJointAverage | wavelet-HHLglcmJointAverage | wavelet-HHHglcmJointAverage | wavelet-LLLglcmJointAverage |
| 36 |  | originalglcmJointEnergy | wavelet-LLHglcmJointEnergy | wavelet-LHLglcmJointEnergy | wavelet-LHHglcmJointEnergy | wavelet-HLLglcmJointEnergy | wavelet-HLHglcmJointEnergy | wavelet-HHLglcmJointEnergy | wavelet-HHHglcmJointEnergy | wavelet-LLLglcmJointEnergy |
| 37 |  | originalglcmJointEntropy | wavelet-LLHglcmJointEntropy | wavelet-LHLglcmJointEntropy | wavelet-LHHglcmJointEntropy | wavelet-HLLglcmJointEntropy | wavelet-HLHglcmJointEntropy | wavelet-HHLglcmJointEntropy | wavelet-HHHglcmJointEntropy | wavelet-LLLglcmJointEntropy |
| 38 |  | originalglcmMCC | wavelet-LLHglcmMCC | wavelet-LHLglcmMCC | wavelet-LHHglcmMCC | wavelet-HLLglcmMCC | wavelet-HLHglcmMCC | wavelet-HHLglcmMCC | wavelet-HHHglcmMCC | wavelet-LLLglcmMCC |
| 39 |  | originalglcmMaximumProbability | wavelet-LLHglcmMaximumProbability | wavelet-LHLglcmMaximumProbability | wavelet-LHHglcmMaximumProbability | wavelet-HLLglcmMaximumProbability | wavelet-HLHglcmMaximumProbability | wavelet-HHLglcmMaximumProbability | wavelet-HHHglcmMaximumProbability | wavelet-LLLglcmMaximumProbability |
| 40 |  | originalglcmSumAverage | wavelet-LLHglcmSumAverage | wavelet-LHLglcmSumAverage | wavelet-LHHglcmSumAverage | wavelet-HLLglcmSumAverage | wavelet-HLHglcmSumAverage | wavelet-HHLglcmSumAverage | wavelet-HHHglcmSumAverage | wavelet-LLLglcmSumAverage |
| 41 |  | originalglcmSumEntropy | wavelet-LLHglcmSumEntropy | wavelet-LHLglcmSumEntropy | wavelet-LHHglcmSumEntropy | wavelet-HLLglcmSumEntropy | wavelet-HLHglcmSumEntropy | wavelet-HHLglcmSumEntropy | wavelet-HHHglcmSumEntropy | wavelet-LLLglcmSumEntropy |
| 42 |  | originalglcmSumSquares | wavelet-LLHglcmSumSquares | wavelet-LHLglcmSumSquares | wavelet-LHHglcmSumSquares | wavelet-HLLglcmSumSquares | wavelet-HLHglcmSumSquares | wavelet-HHLglcmSumSquares | wavelet-HHHglcmSumSquares | wavelet-LLLglcmSumSquares |
| 43 |  | originalgldmDependenceEntropy | wavelet-LLHgldmDependenceEntropy | wavelet-LHLgldmDependenceEntropy | wavelet-LHHgldmDependenceEntropy | wavelet-HLLgldmDependenceEntropy | wavelet-HLHgldmDependenceEntropy | wavelet-HHLgldmDependenceEntropy | wavelet-HHHgldmDependenceEntropy | wavelet-LLLgldmDependenceEntropy |
| 44 |  | originalgldmDependenceNonUniformity | wavelet-LLHgldmDependenceNonUniformity | wavelet-LHLgldmDependenceNonUniformity | wavelet-LHHgldmDependenceNonUniformity | wavelet-HLLgldmDependenceNonUniformity | wavelet-HLHgldmDependenceNonUniformity | wavelet-HHLgldmDependenceNonUniformity | wavelet-HHHgldmDependenceNonUniformity | wavelet-LLLgldmDependenceNonUniformity |
| 45 |  | originalgldmDependenceNonUniformityNormalized | wavelet-LLHgldmDependenceNonUniformityNormalized | wavelet-LHLgldmDependenceNonUniformityNormalized | wavelet-LHHgldmDependenceNonUniformityNormalized | wavelet-HLLgldmDependenceNonUniformityNormalized | wavelet-HLHgldmDependenceNonUniformityNormalized | wavelet-HHLgldmDependenceNonUniformityNormalized | wavelet-HHHgldmDependenceNonUniformityNormalized | wavelet-LLLgldmDependenceNonUniformityNormalized |
| 46 |  | originalgldmDependenceVariance | wavelet-LLHgldmDependenceVariance | wavelet-LHLgldmDependenceVariance | wavelet-LHHgldmDependenceVariance | wavelet-HLLgldmDependenceVariance | wavelet-HLHgldmDependenceVariance | wavelet-HHLgldmDependenceVariance | wavelet-HHHgldmDependenceVariance | wavelet-LLLgldmDependenceVariance |
| 47 |  | originalgldmGrayLevelNonUniformity | wavelet-LLHgldmGrayLevelNonUniformity | wavelet-LHLgldmGrayLevelNonUniformity | wavelet-LHHgldmGrayLevelNonUniformity | wavelet-HLLgldmGrayLevelNonUniformity | wavelet-HLHgldmGrayLevelNonUniformity | wavelet-HHLgldmGrayLevelNonUniformity | wavelet-HHHgldmGrayLevelNonUniformity | wavelet-LLLgldmGrayLevelNonUniformity |
| 48 |  | originalgldmGrayLevelVariance | wavelet-LLHgldmGrayLevelVariance | wavelet-LHLgldmGrayLevelVariance | wavelet-LHHgldmGrayLevelVariance | wavelet-HLLgldmGrayLevelVariance | wavelet-HLHgldmGrayLevelVariance | wavelet-HHLgldmGrayLevelVariance | wavelet-HHHgldmGrayLevelVariance | wavelet-LLLgldmGrayLevelVariance |
| 49 |  | originalgldmHighGrayLevelEmphasis | wavelet-LLHgldmHighGrayLevelEmphasis | wavelet-LHLgldmHighGrayLevelEmphasis | wavelet-LHHgldmHighGrayLevelEmphasis | wavelet-HLLgldmHighGrayLevelEmphasis | wavelet-HLHgldmHighGrayLevelEmphasis | wavelet-HHLgldmHighGrayLevelEmphasis | wavelet-HHHgldmHighGrayLevelEmphasis | wavelet-LLLgldmHighGrayLevelEmphasis |
| 50 |  | originalgldmLargeDependenceEmphasis | wavelet-LLHgldmLargeDependenceEmphasis | wavelet-LHLgldmLargeDependenceEmphasis | wavelet-LHHgldmLargeDependenceEmphasis | wavelet-HLLgldmLargeDependenceEmphasis | wavelet-HLHgldmLargeDependenceEmphasis | wavelet-HHLgldmLargeDependenceEmphasis | wavelet-HHHgldmLargeDependenceEmphasis | wavelet-LLLgldmLargeDependenceEmphasis |
| 51 |  | originalgldmLargeDependenceHighGrayLevelEmphasis | wavelet-LLHgldmLargeDependenceHighGrayLevelEmphasis | wavelet-LHLgldmLargeDependenceHighGrayLevelEmphasis | wavelet-LHHgldmLargeDependenceHighGrayLevelEmphasis | wavelet-HLLgldmLargeDependenceHighGrayLevelEmphasis | wavelet-HLHgldmLargeDependenceHighGrayLevelEmphasis | wavelet-HHLgldmLargeDependenceHighGrayLevelEmphasis | wavelet-HHHgldmLargeDependenceHighGrayLevelEmphasis | wavelet-LLLgldmLargeDependenceHighGrayLevelEmphasis |
| 52 |  | originalgldmLargeDependenceLowGrayLevelEmphasis | wavelet-LLHgldmLargeDependenceLowGrayLevelEmphasis | wavelet-LHLgldmLargeDependenceLowGrayLevelEmphasis | wavelet-LHHgldmLargeDependenceLowGrayLevelEmphasis | wavelet-HLLgldmLargeDependenceLowGrayLevelEmphasis | wavelet-HLHgldmLargeDependenceLowGrayLevelEmphasis | wavelet-HHLgldmLargeDependenceLowGrayLevelEmphasis | wavelet-HHHgldmLargeDependenceLowGrayLevelEmphasis | wavelet-LLLgldmLargeDependenceLowGrayLevelEmphasis |
| 53 |  | originalgldmLowGrayLevelEmphasis | wavelet-LLHgldmLowGrayLevelEmphasis | wavelet-LHLgldmLowGrayLevelEmphasis | wavelet-LHHgldmLowGrayLevelEmphasis | wavelet-HLLgldmLowGrayLevelEmphasis | wavelet-HLHgldmLowGrayLevelEmphasis | wavelet-HHLgldmLowGrayLevelEmphasis | wavelet-HHHgldmLowGrayLevelEmphasis | wavelet-LLLgldmLowGrayLevelEmphasis |
| 54 |  | originalgldmSmallDependenceEmphasis | wavelet-LLHgldmSmallDependenceEmphasis | wavelet-LHLgldmSmallDependenceEmphasis | wavelet-LHHgldmSmallDependenceEmphasis | wavelet-HLLgldmSmallDependenceEmphasis | wavelet-HLHgldmSmallDependenceEmphasis | wavelet-HHLgldmSmallDependenceEmphasis | wavelet-HHHgldmSmallDependenceEmphasis | wavelet-LLLgldmSmallDependenceEmphasis |
| 55 |  | originalgldmSmallDependenceHighGrayLevelEmphasis | wavelet-LLHgldmSmallDependenceHighGrayLevelEmphasis | wavelet-LHLgldmSmallDependenceHighGrayLevelEmphasis | wavelet-LHHgldmSmallDependenceHighGrayLevelEmphasis | wavelet-HLLgldmSmallDependenceHighGrayLevelEmphasis | wavelet-HLHgldmSmallDependenceHighGrayLevelEmphasis | wavelet-HHLgldmSmallDependenceHighGrayLevelEmphasis | wavelet-HHHgldmSmallDependenceHighGrayLevelEmphasis | wavelet-LLLgldmSmallDependenceHighGrayLevelEmphasis |
| 56 |  | originalgldmSmallDependenceLowGrayLevelEmphasis | wavelet-LLHgldmSmallDependenceLowGrayLevelEmphasis | wavelet-LHLgldmSmallDependenceLowGrayLevelEmphasis | wavelet-LHHgldmSmallDependenceLowGrayLevelEmphasis | wavelet-HLLgldmSmallDependenceLowGrayLevelEmphasis | wavelet-HLHgldmSmallDependenceLowGrayLevelEmphasis | wavelet-HHLgldmSmallDependenceLowGrayLevelEmphasis | wavelet-HHHgldmSmallDependenceLowGrayLevelEmphasis | wavelet-LLLgldmSmallDependenceLowGrayLevelEmphasis |
| 57 |  | originalglrlmGrayLevelNonUniformity | wavelet-LLHglrlmGrayLevelNonUniformity | wavelet-LHLglrlmGrayLevelNonUniformity | wavelet-LHHglrlmGrayLevelNonUniformity | wavelet-HLLglrlmGrayLevelNonUniformity | wavelet-HLHglrlmGrayLevelNonUniformity | wavelet-HHLglrlmGrayLevelNonUniformity | wavelet-HHHglrlmGrayLevelNonUniformity | wavelet-LLLglrlmGrayLevelNonUniformity |
| 58 |  | originalglrlmGrayLevelNonUniformityNormalized | wavelet-LLHglrlmGrayLevelNonUniformityNormalized | wavelet-LHLglrlmGrayLevelNonUniformityNormalized | wavelet-LHHglrlmGrayLevelNonUniformityNormalized | wavelet-HLLglrlmGrayLevelNonUniformityNormalized | wavelet-HLHglrlmGrayLevelNonUniformityNormalized | wavelet-HHLglrlmGrayLevelNonUniformityNormalized | wavelet-HHHglrlmGrayLevelNonUniformityNormalized | wavelet-LLLglrlmGrayLevelNonUniformityNormalized |
| 59 |  | originalglrlmGrayLevelVariance | wavelet-LLHglrlmGrayLevelVariance | wavelet-LHLglrlmGrayLevelVariance | wavelet-LHHglrlmGrayLevelVariance | wavelet-HLLglrlmGrayLevelVariance | wavelet-HLHglrlmGrayLevelVariance | wavelet-HHLglrlmGrayLevelVariance | wavelet-HHHglrlmGrayLevelVariance | wavelet-LLLglrlmGrayLevelVariance |
| 60 |  | originalglrlmHighGrayLevelRunEmphasis | wavelet-LLHglrlmHighGrayLevelRunEmphasis | wavelet-LHLglrlmHighGrayLevelRunEmphasis | wavelet-LHHglrlmHighGrayLevelRunEmphasis | wavelet-HLLglrlmHighGrayLevelRunEmphasis | wavelet-HLHglrlmHighGrayLevelRunEmphasis | wavelet-HHLglrlmHighGrayLevelRunEmphasis | wavelet-HHHglrlmHighGrayLevelRunEmphasis | wavelet-LLLglrlmHighGrayLevelRunEmphasis |
| 61 |  | originalglrlmLongRunEmphasis | wavelet-LLHglrlmLongRunEmphasis | wavelet-LHLglrlmLongRunEmphasis | wavelet-LHHglrlmLongRunEmphasis | wavelet-HLLglrlmLongRunEmphasis | wavelet-HLHglrlmLongRunEmphasis | wavelet-HHLglrlmLongRunEmphasis | wavelet-HHHglrlmLongRunEmphasis | wavelet-LLLglrlmLongRunEmphasis |
| 62 |  | originalglrlmLongRunHighGrayLevelEmphasis | wavelet-LLHglrlmLongRunHighGrayLevelEmphasis | wavelet-LHLglrlmLongRunHighGrayLevelEmphasis | wavelet-LHHglrlmLongRunHighGrayLevelEmphasis | wavelet-HLLglrlmLongRunHighGrayLevelEmphasis | wavelet-HLHglrlmLongRunHighGrayLevelEmphasis | wavelet-HHLglrlmLongRunHighGrayLevelEmphasis | wavelet-HHHglrlmLongRunHighGrayLevelEmphasis | wavelet-LLLglrlmLongRunHighGrayLevelEmphasis |
| 63 |  | originalglrlmLongRunLowGrayLevelEmphasis | wavelet-LLHglrlmLongRunLowGrayLevelEmphasis | wavelet-LHLglrlmLongRunLowGrayLevelEmphasis | wavelet-LHHglrlmLongRunLowGrayLevelEmphasis | wavelet-HLLglrlmLongRunLowGrayLevelEmphasis | wavelet-HLHglrlmLongRunLowGrayLevelEmphasis | wavelet-HHLglrlmLongRunLowGrayLevelEmphasis | wavelet-HHHglrlmLongRunLowGrayLevelEmphasis | wavelet-LLLglrlmLongRunLowGrayLevelEmphasis |
| 64 |  | originalglrlmLowGrayLevelRunEmphasis | wavelet-LLHglrlmLowGrayLevelRunEmphasis | wavelet-LHLglrlmLowGrayLevelRunEmphasis | wavelet-LHHglrlmLowGrayLevelRunEmphasis | wavelet-HLLglrlmLowGrayLevelRunEmphasis | wavelet-HLHglrlmLowGrayLevelRunEmphasis | wavelet-HHLglrlmLowGrayLevelRunEmphasis | wavelet-HHHglrlmLowGrayLevelRunEmphasis | wavelet-LLLglrlmLowGrayLevelRunEmphasis |
| 65 |  | originalglrlmRunEntropy | wavelet-LLHglrlmRunEntropy | wavelet-LHLglrlmRunEntropy | wavelet-LHHglrlmRunEntropy | wavelet-HLLglrlmRunEntropy | wavelet-HLHglrlmRunEntropy | wavelet-HHLglrlmRunEntropy | wavelet-HHHglrlmRunEntropy | wavelet-LLLglrlmRunEntropy |
| 66 |  | originalglrlmRunLengthNonUniformity | wavelet-LLHglrlmRunLengthNonUniformity | wavelet-LHLglrlmRunLengthNonUniformity | wavelet-LHHglrlmRunLengthNonUniformity | wavelet-HLLglrlmRunLengthNonUniformity | wavelet-HLHglrlmRunLengthNonUniformity | wavelet-HHLglrlmRunLengthNonUniformity | wavelet-HHHglrlmRunLengthNonUniformity | wavelet-LLLglrlmRunLengthNonUniformity |
| 67 |  | originalglrlmRunLengthNonUniformityNormalized | wavelet-LLHglrlmRunLengthNonUniformityNormalized | wavelet-LHLglrlmRunLengthNonUniformityNormalized | wavelet-LHHglrlmRunLengthNonUniformityNormalized | wavelet-HLLglrlmRunLengthNonUniformityNormalized | wavelet-HLHglrlmRunLengthNonUniformityNormalized | wavelet-HHLglrlmRunLengthNonUniformityNormalized | wavelet-HHHglrlmRunLengthNonUniformityNormalized | wavelet-LLLglrlmRunLengthNonUniformityNormalized |
| 68 |  | originalglrlmRunPercentage | wavelet-LLHglrlmRunPercentage | wavelet-LHLglrlmRunPercentage | wavelet-LHHglrlmRunPercentage | wavelet-HLLglrlmRunPercentage | wavelet-HLHglrlmRunPercentage | wavelet-HHLglrlmRunPercentage | wavelet-HHHglrlmRunPercentage | wavelet-LLLglrlmRunPercentage |
| 69 |  | originalglrlmRunVariance | wavelet-LLHglrlmRunVariance | wavelet-LHLglrlmRunVariance | wavelet-LHHglrlmRunVariance | wavelet-HLLglrlmRunVariance | wavelet-HLHglrlmRunVariance | wavelet-HHLglrlmRunVariance | wavelet-HHHglrlmRunVariance | wavelet-LLLglrlmRunVariance |
| 70 |  | originalglrlmShortRunEmphasis | wavelet-LLHglrlmShortRunEmphasis | wavelet-LHLglrlmShortRunEmphasis | wavelet-LHHglrlmShortRunEmphasis | wavelet-HLLglrlmShortRunEmphasis | wavelet-HLHglrlmShortRunEmphasis | wavelet-HHLglrlmShortRunEmphasis | wavelet-HHHglrlmShortRunEmphasis | wavelet-LLLglrlmShortRunEmphasis |
| 71 |  | originalglrlmShortRunHighGrayLevelEmphasis | wavelet-LLHglrlmShortRunHighGrayLevelEmphasis | wavelet-LHLglrlmShortRunHighGrayLevelEmphasis | wavelet-LHHglrlmShortRunHighGrayLevelEmphasis | wavelet-HLLglrlmShortRunHighGrayLevelEmphasis | wavelet-HLHglrlmShortRunHighGrayLevelEmphasis | wavelet-HHLglrlmShortRunHighGrayLevelEmphasis | wavelet-HHHglrlmShortRunHighGrayLevelEmphasis | wavelet-LLLglrlmShortRunHighGrayLevelEmphasis |
| 72 |  | originalglrlmShortRunLowGrayLevelEmphasis | wavelet-LLHglrlmShortRunLowGrayLevelEmphasis | wavelet-LHLglrlmShortRunLowGrayLevelEmphasis | wavelet-LHHglrlmShortRunLowGrayLevelEmphasis | wavelet-HLLglrlmShortRunLowGrayLevelEmphasis | wavelet-HLHglrlmShortRunLowGrayLevelEmphasis | wavelet-HHLglrlmShortRunLowGrayLevelEmphasis | wavelet-HHHglrlmShortRunLowGrayLevelEmphasis | wavelet-LLLglrlmShortRunLowGrayLevelEmphasis |
| 73 |  | originalglszmGrayLevelNonUniformity | wavelet-LLHglszmGrayLevelNonUniformity | wavelet-LHLglszmGrayLevelNonUniformity | wavelet-LHHglszmGrayLevelNonUniformity | wavelet-HLLglszmGrayLevelNonUniformity | wavelet-HLHglszmGrayLevelNonUniformity | wavelet-HHLglszmGrayLevelNonUniformity | wavelet-HHHglszmGrayLevelNonUniformity | wavelet-LLLglszmGrayLevelNonUniformity |
| 74 |  | originalglszmGrayLevelNonUniformityNormalized | wavelet-LLHglszmGrayLevelNonUniformityNormalized | wavelet-LHLglszmGrayLevelNonUniformityNormalized | wavelet-LHHglszmGrayLevelNonUniformityNormalized | wavelet-HLLglszmGrayLevelNonUniformityNormalized | wavelet-HLHglszmGrayLevelNonUniformityNormalized | wavelet-HHLglszmGrayLevelNonUniformityNormalized | wavelet-HHHglszmGrayLevelNonUniformityNormalized | wavelet-LLLglszmGrayLevelNonUniformityNormalized |
| 75 |  | originalglszmGrayLevelVariance | wavelet-LLHglszmGrayLevelVariance | wavelet-LHLglszmGrayLevelVariance | wavelet-LHHglszmGrayLevelVariance | wavelet-HLLglszmGrayLevelVariance | wavelet-HLHglszmGrayLevelVariance | wavelet-HHLglszmGrayLevelVariance | wavelet-HHHglszmGrayLevelVariance | wavelet-LLLglszmGrayLevelVariance |
| 76 |  | originalglszmHighGrayLevelZoneEmphasis | wavelet-LLHglszmHighGrayLevelZoneEmphasis | wavelet-LHLglszmHighGrayLevelZoneEmphasis | wavelet-LHHglszmHighGrayLevelZoneEmphasis | wavelet-HLLglszmHighGrayLevelZoneEmphasis | wavelet-HLHglszmHighGrayLevelZoneEmphasis | wavelet-HHLglszmHighGrayLevelZoneEmphasis | wavelet-HHHglszmHighGrayLevelZoneEmphasis | wavelet-LLLglszmHighGrayLevelZoneEmphasis |
| 77 |  | originalglszmLargeAreaEmphasis | wavelet-LLHglszmLargeAreaEmphasis | wavelet-LHLglszmLargeAreaEmphasis | wavelet-LHHglszmLargeAreaEmphasis | wavelet-HLLglszmLargeAreaEmphasis | wavelet-HLHglszmLargeAreaEmphasis | wavelet-HHLglszmLargeAreaEmphasis | wavelet-HHHglszmLargeAreaEmphasis | wavelet-LLLglszmLargeAreaEmphasis |
| 78 |  | originalglszmLargeAreaHighGrayLevelEmphasis | wavelet-LLHglszmLargeAreaHighGrayLevelEmphasis | wavelet-LHLglszmLargeAreaHighGrayLevelEmphasis | wavelet-LHHglszmLargeAreaHighGrayLevelEmphasis | wavelet-HLLglszmLargeAreaHighGrayLevelEmphasis | wavelet-HLHglszmLargeAreaHighGrayLevelEmphasis | wavelet-HHLglszmLargeAreaHighGrayLevelEmphasis | wavelet-HHHglszmLargeAreaHighGrayLevelEmphasis | wavelet-LLLglszmLargeAreaHighGrayLevelEmphasis |
| 79 |  | originalglszmLargeAreaLowGrayLevelEmphasis | wavelet-LLHglszmLargeAreaLowGrayLevelEmphasis | wavelet-LHLglszmLargeAreaLowGrayLevelEmphasis | wavelet-LHHglszmLargeAreaLowGrayLevelEmphasis | wavelet-HLLglszmLargeAreaLowGrayLevelEmphasis | wavelet-HLHglszmLargeAreaLowGrayLevelEmphasis | wavelet-HHLglszmLargeAreaLowGrayLevelEmphasis | wavelet-HHHglszmLargeAreaLowGrayLevelEmphasis | wavelet-LLLglszmLargeAreaLowGrayLevelEmphasis |
| 80 |  | originalglszmLowGrayLevelZoneEmphasis | wavelet-LLHglszmLowGrayLevelZoneEmphasis | wavelet-LHLglszmLowGrayLevelZoneEmphasis | wavelet-LHHglszmLowGrayLevelZoneEmphasis | wavelet-HLLglszmLowGrayLevelZoneEmphasis | wavelet-HLHglszmLowGrayLevelZoneEmphasis | wavelet-HHLglszmLowGrayLevelZoneEmphasis | wavelet-HHHglszmLowGrayLevelZoneEmphasis | wavelet-LLLglszmLowGrayLevelZoneEmphasis |
| 81 |  | originalglszmSizeZoneNonUniformity | wavelet-LLHglszmSizeZoneNonUniformity | wavelet-LHLglszmSizeZoneNonUniformity | wavelet-LHHglszmSizeZoneNonUniformity | wavelet-HLLglszmSizeZoneNonUniformity | wavelet-HLHglszmSizeZoneNonUniformity | wavelet-HHLglszmSizeZoneNonUniformity | wavelet-HHHglszmSizeZoneNonUniformity | wavelet-LLLglszmSizeZoneNonUniformity |
| 82 |  | originalglszmSizeZoneNonUniformityNormalized | wavelet-LLHglszmSizeZoneNonUniformityNormalized | wavelet-LHLglszmSizeZoneNonUniformityNormalized | wavelet-LHHglszmSizeZoneNonUniformityNormalized | wavelet-HLLglszmSizeZoneNonUniformityNormalized | wavelet-HLHglszmSizeZoneNonUniformityNormalized | wavelet-HHLglszmSizeZoneNonUniformityNormalized | wavelet-HHHglszmSizeZoneNonUniformityNormalized | wavelet-LLLglszmSizeZoneNonUniformityNormalized |
| 83 |  | originalglszmSmallAreaEmphasis | wavelet-LLHglszmSmallAreaEmphasis | wavelet-LHLglszmSmallAreaEmphasis | wavelet-LHHglszmSmallAreaEmphasis | wavelet-HLLglszmSmallAreaEmphasis | wavelet-HLHglszmSmallAreaEmphasis | wavelet-HHLglszmSmallAreaEmphasis | wavelet-HHHglszmSmallAreaEmphasis | wavelet-LLLglszmSmallAreaEmphasis |
| 84 |  | originalglszmSmallAreaHighGrayLevelEmphasis | wavelet-LLHglszmSmallAreaHighGrayLevelEmphasis | wavelet-LHLglszmSmallAreaHighGrayLevelEmphasis | wavelet-LHHglszmSmallAreaHighGrayLevelEmphasis | wavelet-HLLglszmSmallAreaHighGrayLevelEmphasis | wavelet-HLHglszmSmallAreaHighGrayLevelEmphasis | wavelet-HHLglszmSmallAreaHighGrayLevelEmphasis | wavelet-HHHglszmSmallAreaHighGrayLevelEmphasis | wavelet-LLLglszmSmallAreaHighGrayLevelEmphasis |
| 85 |  | originalglszmSmallAreaLowGrayLevelEmphasis | wavelet-LLHglszmSmallAreaLowGrayLevelEmphasis | wavelet-LHLglszmSmallAreaLowGrayLevelEmphasis | wavelet-LHHglszmSmallAreaLowGrayLevelEmphasis | wavelet-HLLglszmSmallAreaLowGrayLevelEmphasis | wavelet-HLHglszmSmallAreaLowGrayLevelEmphasis | wavelet-HHLglszmSmallAreaLowGrayLevelEmphasis | wavelet-HHHglszmSmallAreaLowGrayLevelEmphasis | wavelet-LLLglszmSmallAreaLowGrayLevelEmphasis |
| 86 |  | originalglszmZoneEntropy | wavelet-LLHglszmZoneEntropy | wavelet-LHLglszmZoneEntropy | wavelet-LHHglszmZoneEntropy | wavelet-HLLglszmZoneEntropy | wavelet-HLHglszmZoneEntropy | wavelet-HHLglszmZoneEntropy | wavelet-HHHglszmZoneEntropy | wavelet-LLLglszmZoneEntropy |
| 87 |  | originalglszmZonePercentage | wavelet-LLHglszmZonePercentage | wavelet-LHLglszmZonePercentage | wavelet-LHHglszmZonePercentage | wavelet-HLLglszmZonePercentage | wavelet-HLHglszmZonePercentage | wavelet-HHLglszmZonePercentage | wavelet-HHHglszmZonePercentage | wavelet-LLLglszmZonePercentage |
| 88 |  | originalglszmZoneVariance | wavelet-LLHglszmZoneVariance | wavelet-LHLglszmZoneVariance | wavelet-LHHglszmZoneVariance | wavelet-HLLglszmZoneVariance | wavelet-HLHglszmZoneVariance | wavelet-HHLglszmZoneVariance | wavelet-HHHglszmZoneVariance | wavelet-LLLglszmZoneVariance |
| 89 |  | originalngtdmBusyness | wavelet-LLHngtdmBusyness | wavelet-LHLngtdmBusyness | wavelet-LHHngtdmBusyness | wavelet-HLLngtdmBusyness | wavelet-HLHngtdmBusyness | wavelet-HHLngtdmBusyness | wavelet-HHHngtdmBusyness | wavelet-LLLngtdmBusyness |
| 90 |  | originalngtdmCoarseness | wavelet-LLHngtdmCoarseness | wavelet-LHLngtdmCoarseness | wavelet-LHHngtdmCoarseness | wavelet-HLLngtdmCoarseness | wavelet-HLHngtdmCoarseness | wavelet-HHLngtdmCoarseness | wavelet-HHHngtdmCoarseness | wavelet-LLLngtdmCoarseness |
| 91 |  | originalngtdmComplexity | wavelet-LLHngtdmComplexity | wavelet-LHLngtdmComplexity | wavelet-LHHngtdmComplexity | wavelet-HLLngtdmComplexity | wavelet-HLHngtdmComplexity | wavelet-HHLngtdmComplexity | wavelet-HHHngtdmComplexity | wavelet-LLLngtdmComplexity |
| 92 |  | originalngtdmContrast | wavelet-LLHngtdmContrast | wavelet-LHLngtdmContrast | wavelet-LHHngtdmContrast | wavelet-HLLngtdmContrast | wavelet-HLHngtdmContrast | wavelet-HHLngtdmContrast | wavelet-HHHngtdmContrast | wavelet-LLLngtdmContrast |
| 93 |  | originalngtdmStrength | wavelet-LLHngtdmStrength | wavelet-LHLngtdmStrength | wavelet-LHHngtdmStrength | wavelet-HLLngtdmStrength | wavelet-HLHngtdmStrength | wavelet-HHLngtdmStrength | wavelet-HHHngtdmStrength | wavelet-LLLngtdmStrength |

**Supplementary Material Figure S1.** (a) LASSO regression of short-term efficacy model. (b) LASSO regression of iPFS model


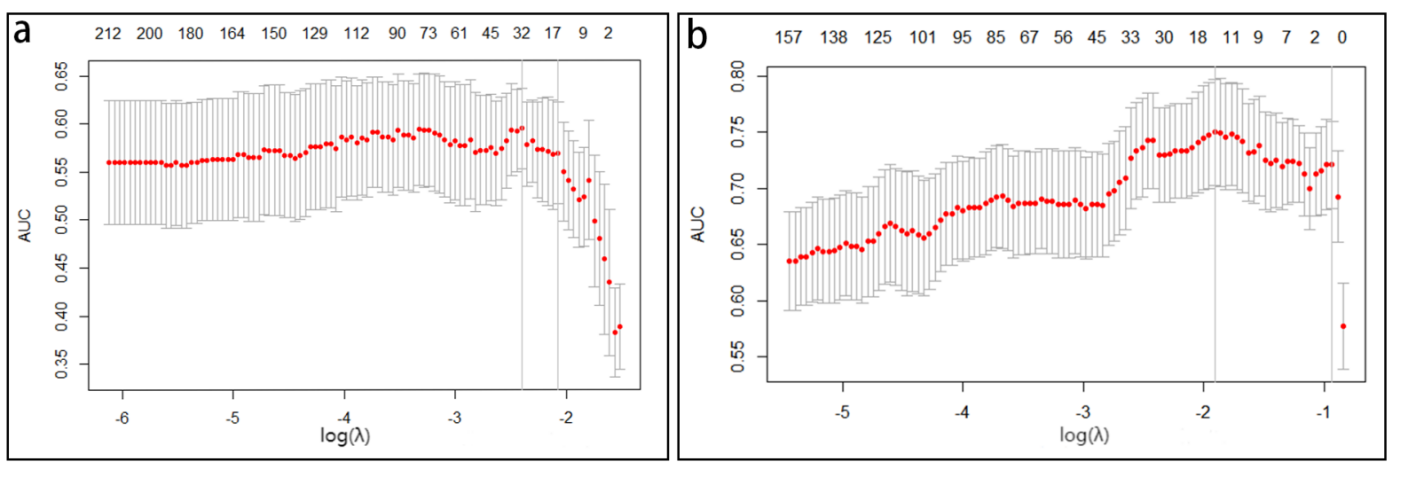


**Supplementary Material Figure S2.** (a) R-plot algorithm for the relationship between the region-of-interest image filtration process and short-term efficacy by 10-fold cross-validation LASSO method. The gray line represents the value of log (λ) when selecting the best number of features. Log (λ) = -2.1, best number=16. (b) R-plot algorithm for the relationship between the region-of-interest image filtration process and iPFS. Log (λ) = -1.9, best number = 13.


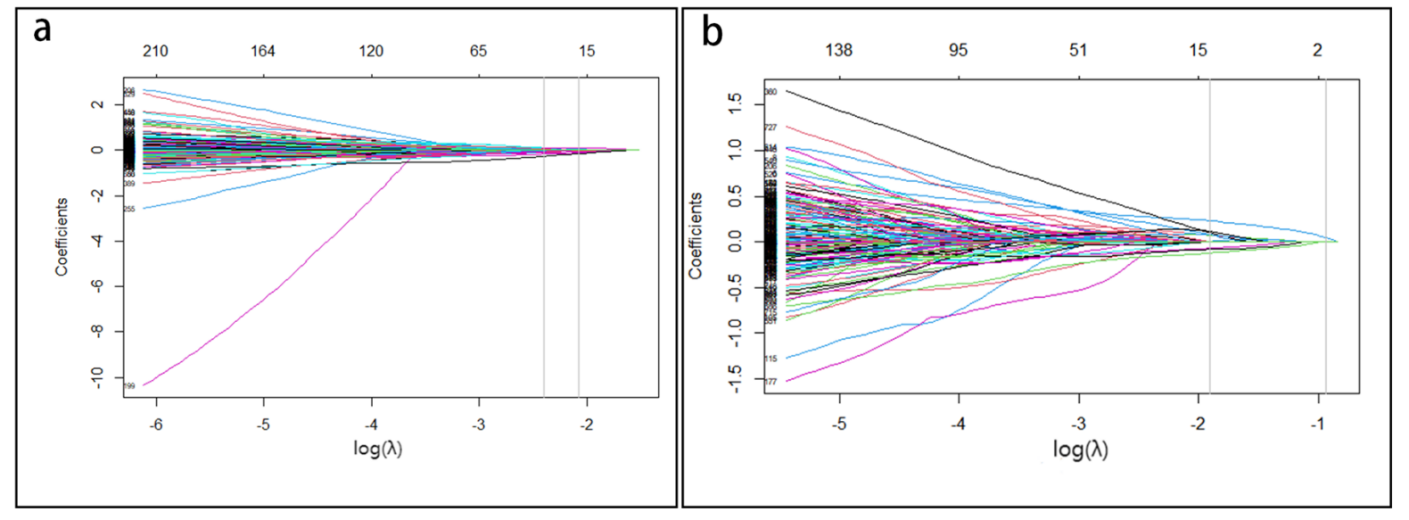


**Supplementary Material Figure S3.** (a) ROC curves of training cohort and validation cohort constructed according to radiomics signature of the short-term efficacy model. (b) ROC curves of training cohort and validation cohort constructed according to radiomics signature of the iPFS model.


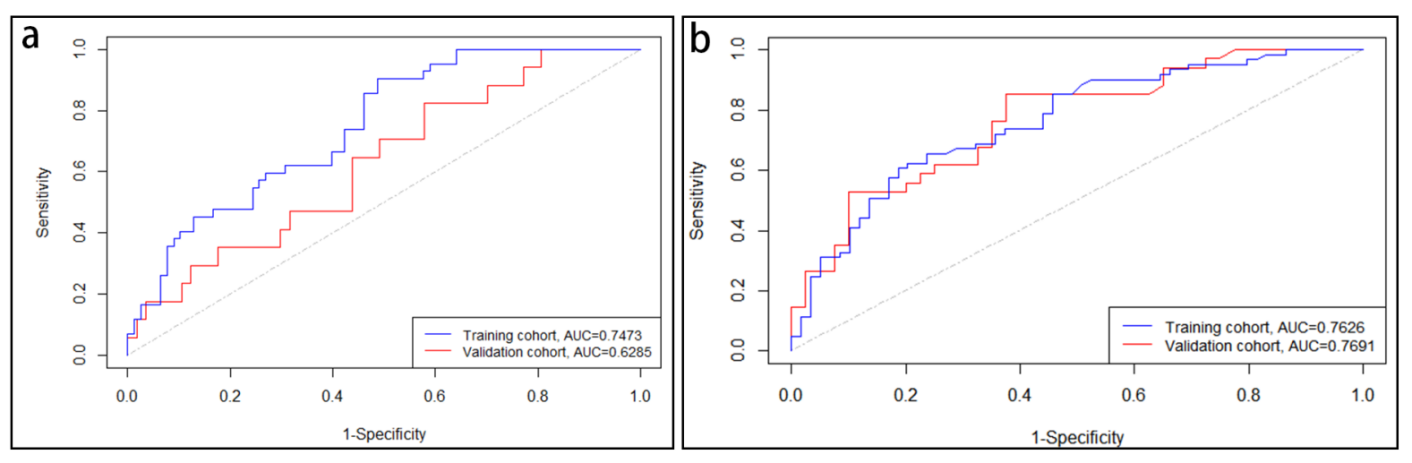


**Supplementary Material Figure S4.** (a) Clinical nomogram of short-term efficacy model. (b) Radiomics nomogram of shortterm efficacy model. (c) Clinical nomogram of iPFS model. (d) Radiomics nomogram of iPFS model.

**
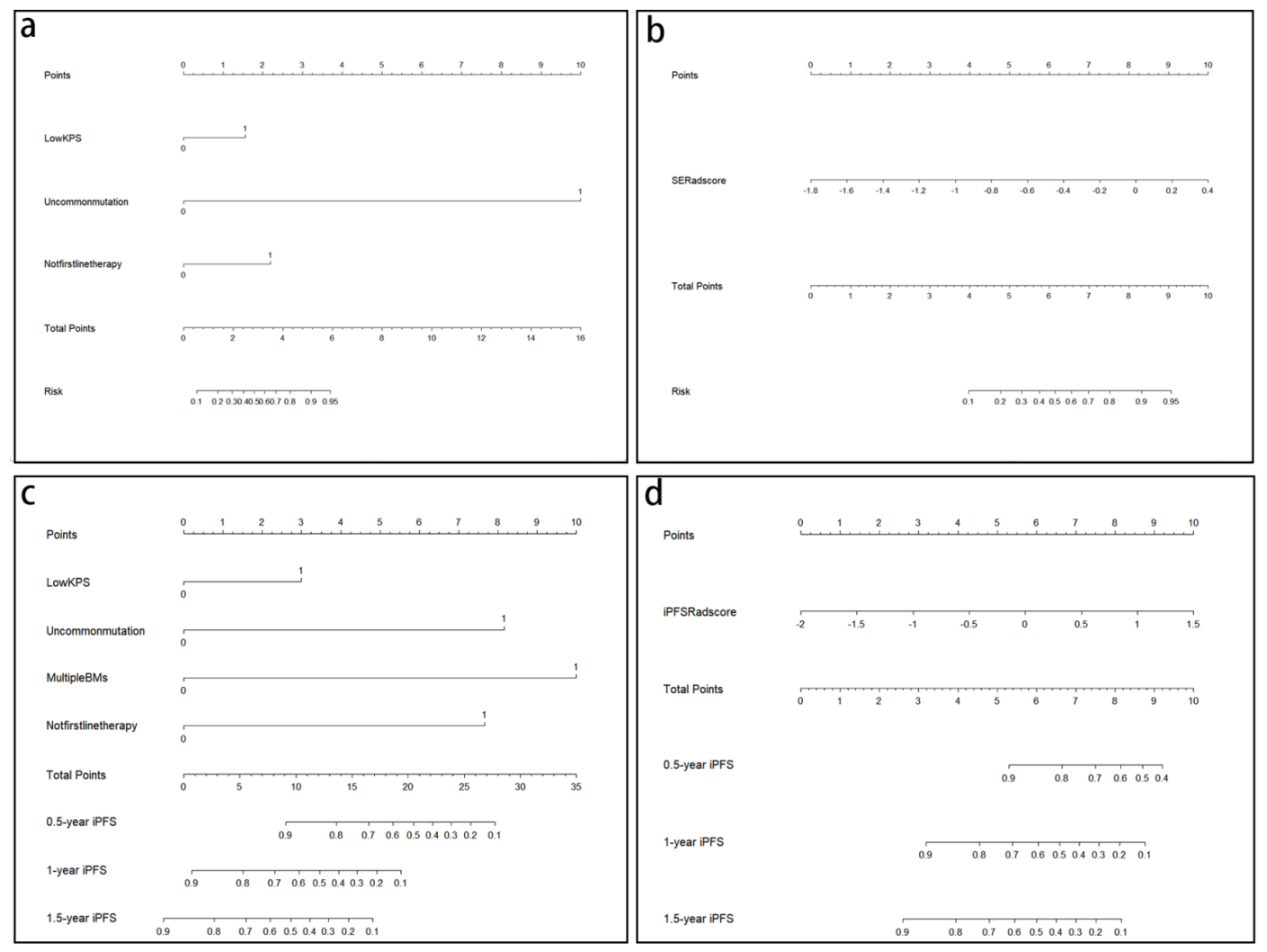
**

**Supplementary Material Figure S5.** (a) Calibration curve of iPFS clinical nomogram. (b) Calibration curve of iPFS radiomics nomogram. (c) Calibration curve of short-term efficacy clinical nomogram. (d) Calibration curve of short-term efficacy radiomics nomogram.


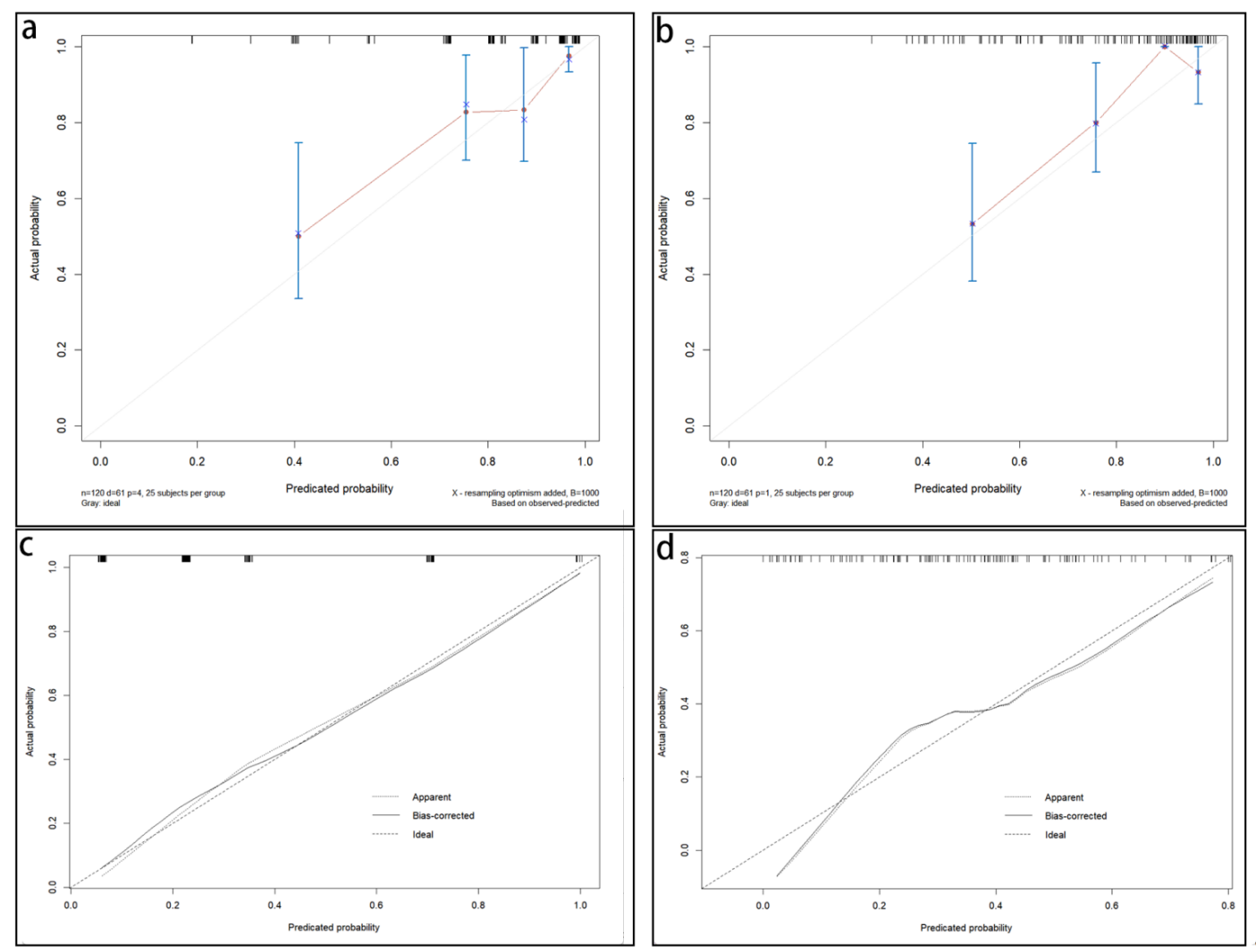

Supplement: Supplementary file 1 — Supplementary Material 1. [file 12885_2024_12121_MOESM1_ESM.docx]
